# PhenoSpD: an integrated toolkit for phenotypic correlation estimation and multiple testing correction using GWAS summary statistics

**DOI:** 10.1093/gigascience/giy090

**Published:** 2018-08-24

**Authors:** Jie Zheng, Tom G Richardson, Louise A C Millard, Gibran Hemani, Benjamin L Elsworth, Christopher A Raistrick, Bjarni Vilhjalmsson, Benjamin M Neale, Philip C Haycock, George Davey Smith, Tom R Gaunt

**Affiliations:** 1 MRC Integrative Epidemiology Unit, University of Bristol, Oakfield House, Bristol, BS8 2BN, UK; 2 Intelligent Systems Laboratory, University of Bristol, Tyndall Ave, Bristol, BS8 1TH, UK; 3 Åarhus Center for Bioinformatics BIRC, Aarhus University, Nordre Ringgade, 1,8000, Aarhus C, Denmark; 4 Program in Medical and Population Genetics, Broad Institute of MIT and Harvard, 415 Main St, Cambridge, MA, 02142, USA; 5 Analytical and Translational Genetics Unit, Department of Medicine, Massachusetts General Hospital and Harvard Medical School, Boston, MA, 02114, USA

**Keywords:** Phenotypic correlation, multiple testing, LD score regression, MetaCCA, Genome-wide association study, Summary Statistics

## Abstract

**Background:**

Identifying phenotypic correlations between complex traits and diseases can provide useful etiological insights. Restricted access to much individual-level phenotype data makes it difficult to estimate large-scale phenotypic correlation across the human phenome. Two state-of-the-art methods, metaCCA and LD score regression, provide an alternative approach to estimate phenotypic correlation using only genome-wide association study (GWAS) summary results.

**Results:**

Here, we present an integrated R toolkit, PhenoSpD, to use LD score regression to estimate phenotypic correlations using GWAS summary statistics and to utilize the estimated phenotypic correlations to inform correction of multiple testing for complex human traits using the spectral decomposition of matrices (SpD). The simulations suggest that it is possible to identify nonindependence of phenotypes using samples with partial overlap; as overlap decreases, the estimated phenotypic correlations will attenuate toward zero and multiple testing correction will be more stringent than in perfectly overlapping samples. Also, in contrast to LD score regression, metaCCA will provide approximate genetic correlations rather than phenotypic correlation, which limits its application for multiple testing correction. In a case study, PhenoSpD using UK Biobank GWAS results suggested 399.6 independent tests among 487 human traits, which is close to the 352.4 independent tests estimated using true phenotypic correlation. We further applied PhenoSpD to an estimated 5,618 pair-wise phenotypic correlations among 107 metabolites using GWAS summary statistics from Kettunen's publication and PhenoSpD suggested the equivalent of 33.5 independent tests for these metabolites.

**Conclusions:**

PhenoSpD extends the use of summary-level results, providing a simple and conservative way to reduce dimensionality for complex human traits using GWAS summary statistics. This is particularly valuable in the age of large-scale biobank and consortia studies, where GWAS results are much more accessible than individual-level data.

## Introduction

Phenotypic correlations between complex human traits and diseases can provide useful etiological insights into the understanding of mechanisms across the human phenome. However, a lack of individual-level phenotype data makes it difficult to estimate the phenotypic correlations between human traits and diseases. Fortunately, we are now in the post genome-wide association study (GWAS) era, in which many GWAS summary results are openly accessible for a large number of human diseases and traits [1]. It can therefore be valuable to use these genetic association summary statistics to reconstruct total phenotypic correlations across the human phenome. The key assumptions here are that phenotypic correlation comprises both genetic and nongenetic (environmental) components and that the genetic association information is able to capture both genetic and nongenetic components of the phenotypic correlation [2].

Here, we consider two methods that can be used (but were not designed) to estimate phenotypic correlations using GWAS summary statistics as by-products of the main purposes of those methods. First, MetaCCA [3] is a multivariate meta-analysis tool that allows multivariate representation of both genotype and phenotype. As a by-product, metaCCA estimates the phenotypic correlation between two traits based on a Pearson correlation between two univariable regression coefficients (betas) across a set of genetic variants. Second, bivariate linkage disequilibrium (LD) score regression [2] is a state-of-the-art approach to estimate genetic correlations between a pair of traits. As a consequence, the bivariate LD score regression approach allows estimation of phenotypic correlation among the overlapping samples of two GWASs. Assuming the genetic and nongenetic components of two phenotypes are independent, the genetic covariance matrix (built up by the beta coefficients of the genetic association test) will capture the genetic effects, while the error covariance matrix (built up by the error term of the genetic association test) will capture the environmental (nongenetic) effects. Using a bivariate LD score regression model, we are able to capture both (genetic correlation will be represented by the slope of the regression model and phenotypic correlation will be represented by the intercept of the regression model) [2].

Large-scale genetic association databases such as MR-Base [4] and LD Hub [5] have harmonized GWAS summary-level results for roughly 1,700 human traits. This provides a timely opportunity to estimate the phenotypic correlation structure across a wide range of high-dimensional, complex molecular traits, such as metabolites, that are potentially highly correlated. Bonferroni correction would markedly overcorrect for the inflated false-positive rate in such correlated datasets, resulting in a reduction in power. An appropriate method to correct for multiple testing among human traits and diseases is the spectral decomposition of matrices (SpD) [6, 7]. Here, we combine LD score regression with SpD to estimate the number of independent tests using only summary-level GWAS data.

## Methods

### Overview of PhenoSpD

**Figure 1:**
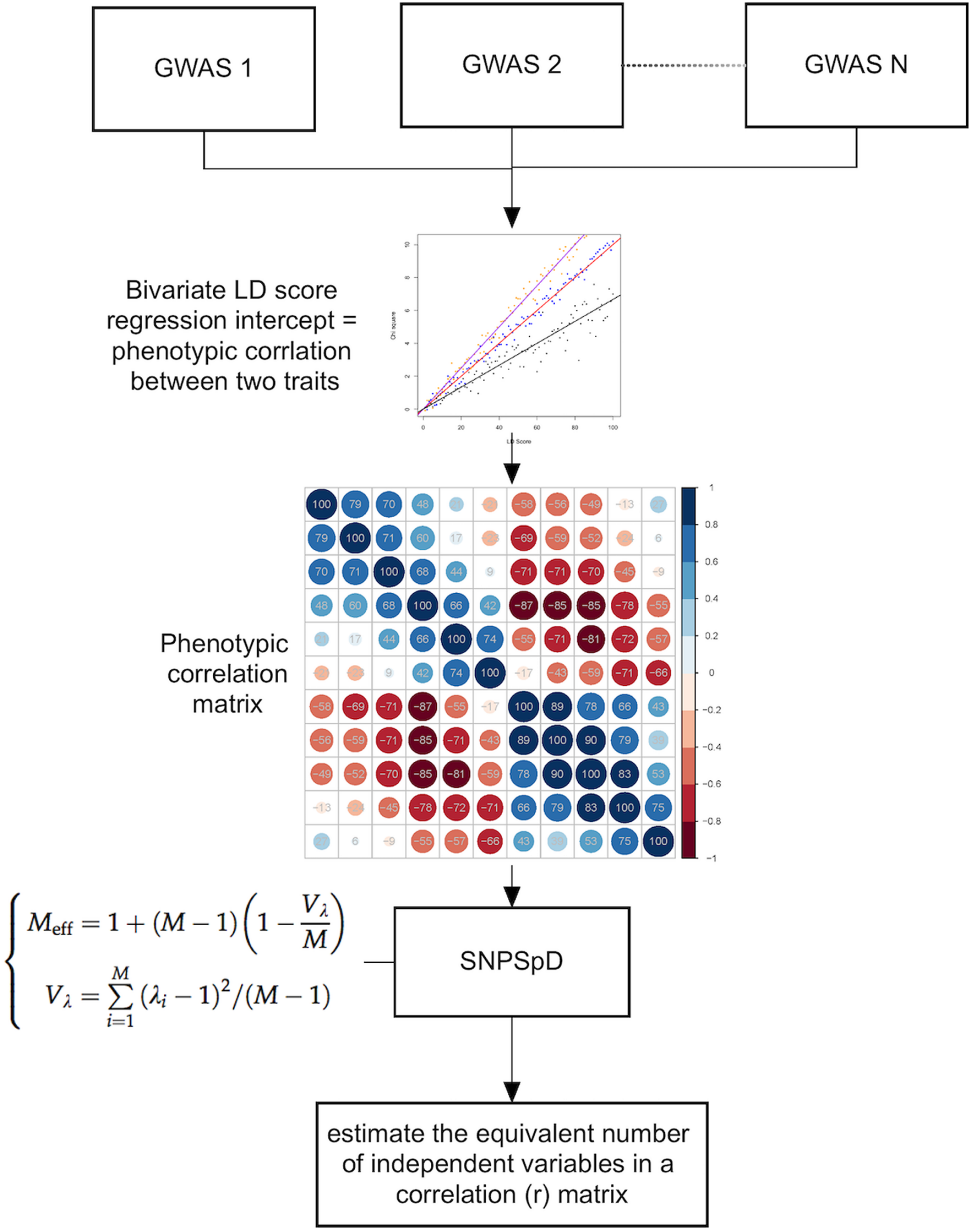
Flowchart of PhenoSpD.

Fig. 1 illustrates the key steps of the proposed pipeline, PhenoSpD, as follows: (1) harmonize GWAS summary results from the same sample; (2) apply the harmonized GWAS results to LD score regression to estimate the phenotypic correlation matrix of the traits; and (3) apply the SpD approach to the phenotypic correlation matrix and estimate the number of independent variables among the traits.

### Simulation of phenotypic correlation estimation

**Figure 2:**
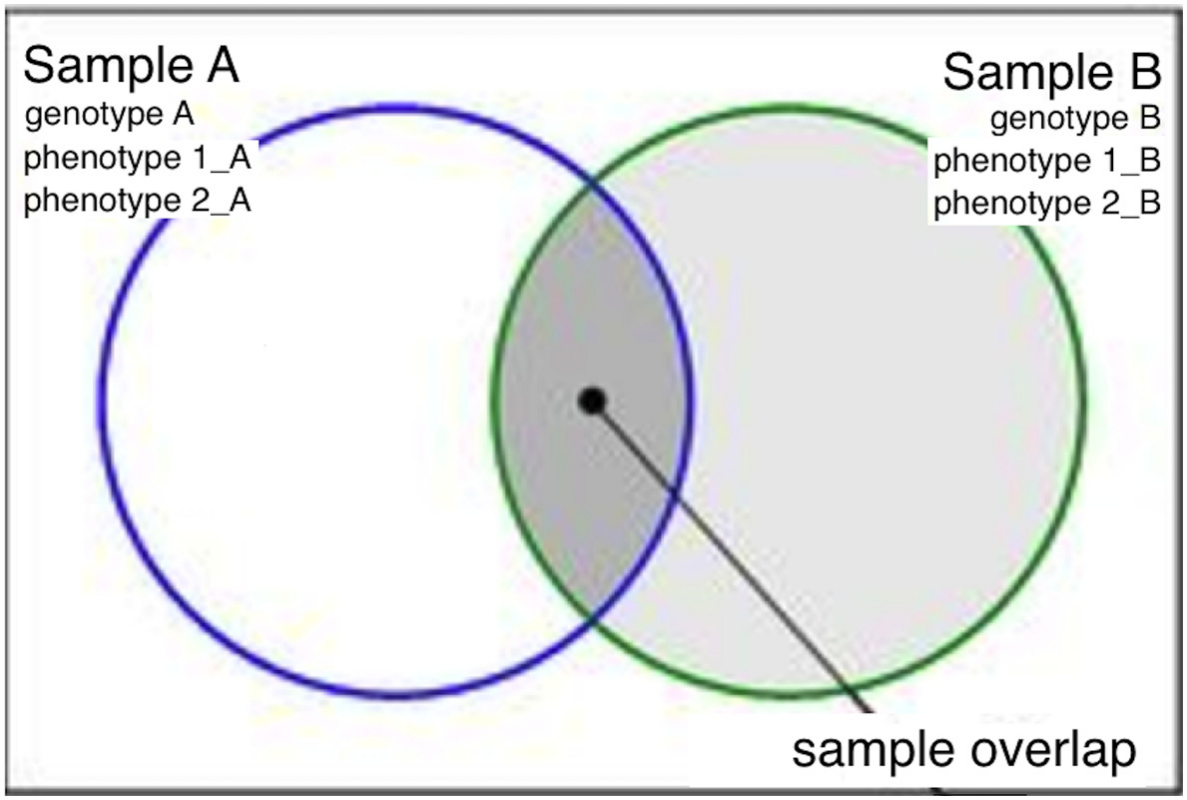
Demonstration of the simulation. For two samples A and B, we simulated the genotype data and phenotype data of two correlated human traits, phenotype 1 and phenotype 2. The sample overlap between sample A and sample B ranged from 10% to 90% in this simulation.

First, we simulated the influence of the number of single nucleotide polymorphisms (SNPs), sample sizes of two GWASs, and sample overlap between two GWASs on the accuracy of the phenotypic correlation estimation. As shown in Fig. 2, we first created two samples A and B with different numbers of individuals (from 300 to  10,000 individuals), where the sample overlap between sample A and B ranged from 10% to 90%. We assumed complex human traits were influenced by both genetic and environmental factors, so we simulated the phenotype data of two correlated human traits (phenotype 1 and phenotype 2 with a phenotypic correlation of –0.7) based on varying numbers of genetic factors (ranging from 10 to  10,000 SNPs), different LD structure (r^2^ between 0 and 0.9), and 100 environmental factors. We then assigned the phenotypic correlation to its genetic and environmental components, each of which explained 10% to 90% of the total phenotypic variance. These genetic and environmental components were further assigned to each of the genetic and environmental factors in the model randomly. We also simulated two extreme cases where either the genetic or environmental components dominate the phenotypic correlation. After simulating the two phenotypic traits and the genotypic data in samples A and B, we conducted four GWASs (GWASs of phenotype 1 in samples A and B; GWASs of phenotype 2 in samples A and B) and recorded the summary statistics of these GWASs. To measure the accuracy of phenotypic correlation using GWAS summary statistics, we (1) calculated the observational phenotypic correlation (the Pearson correlation) between trait 1 and trait 2 in samples A and B separately and (2) estimated the phenotypic correlation between trait 1 and trait 2 in the overlapped samples using both metaCCA and LD score regression. We simulated step (2) 100 times and estimated the mean and standard deviation of the estimated phenotypic correlations. Finally, we compared the estimated phenotypic correlation with the observational phenotypic correlation and recorded the deviation between observed and estimated correlations. To demonstrate the simulation systematically, we explored the influence of the following properties: sample size; sample overlap; unbalanced sample size in samples A and B; number of SNPs; and LD. The R script for this simulation is provided as a Supplementary File (simulation.R).

### Validation of phenotypic correlation estimation using real GWAS data

We further tested the accuracy of the phenotypic correlation estimation using GWAS summary statistics of 487 traits from the UK Biobank [8] (Supplementary Table S2). We calculated the observational phenotypic correlation using the actual phenotype data (Supplementary Table S4), which was used as a benchmark to evaluate the accuracy of our phenotypic correlation estimates using LD score regression.

In addition, we tested whether the number of causal variants (which are tagged by the genetic association signals) may affect the accuracy of the phenotypic correlation using four pairs of metabolites from Shin et al. [9]. The four pairs of metabolites were selected because they have a wide range of observed phenotypic correlation from 0.2 to 0.85. To validate the accuracy, we compared the observed phenotypic correlation with the phenotypic correlation estimated by LD score regression. To consider the number of causal variants in this validation, we set up eight groups of SNPs based on their effects on the traits. The eight groups were all GWAS SNPs; SNPs with Chi square statistics (square of Z scores) smaller than 40; SNPs with X^2^ <30; SNPs with X^2^ <20; SNPs with X^2^ <10; SNPs with X^2^ <3.84; SNPs with X^2^ <2.69; and SNPs with X^2^ <1. In other words, we progressively reduced the number of casual variants from the model and evaluated the impact of this on the accuracy of the phenotypic correlation estimation.

Based on the simulation and real case validation, we listed our traits selection criteria in Supplementary Table S1.

### Estimating the phenotypic correlations

Within our GWAS summary results database containing roughly 1,700 human traits, we selected 107 metabolites from Kettunen et al.[10] as a real case application since these complex molecular traits are potentially highly correlated. We then applied LD score regression to these 107 metabolites to estimate the phenotypic correlation matrix (Supplementary Table S3), which meets the suggested minimum parameters of the LD score regression method (traits with large sample size [e.g., *N* >5000], good SNP coverage [e.g., number of SNPs  >200,000], and heritable [e.g., Z score of the SNP heritability >2]).

### Multiple testing correction for human traits

We applied the SpD approach to estimate the number of independent tests among the 107 metabolites and 487 UK Biobank traits. The observed phenotypic correlations and correlations estimated by LD score regression were used as input for the SpD approach. We implemented the R code of the well-known method SNPSpD [6, 7] to estimate the number of independent traits using the phenotypic correlation matrix as input (Fig.1). The output of the SpD function is the estimated number of independent tests.

## Results

### Evaluation of phenotypic correlation estimation using simulated and real GWAS summary data

Tables 1 and 2 show the influence of changing various parameters on the accuracy of the phenotypic correlation estimation for metaCCA and LD score regression, respectively. Our general observations from the simulation are that since the genetic association information is able to capture both genetic and nongenetic components of the phenotypic correlation, we can estimate such correlation for any human trait, even for nonheritable traits. Also, we should apply LD score regression to estimate phenotypic correlation in a one-sample setting (i.e., where all GWAS are performed in the same sample). It is possible to identify nonindependence of phenotypes using GWAS results from samples with only a partial overlap; however, as overlap decreases, correlations will attenuate toward zero. In addition, metaCCA will provide approximate genetic correlations rather than phenotypic correlation, which limits its application in our approach to evaluating multiple testing.

**Table 1:** The influence of genetic and environmental components on phenotypic correlation estimation using metaCCA

Model	N_ind_A	N_ind_B	N_overlap	Overlap_%	N_SNPs	SNP_region	N_EnvF	Genetic%	N_simu	Obs_rp	rG	rE	Est_rp
Genetic_Env_components 1	5000	5000	5000	100	1000	SNPs across the genome	1000	0	100	0.497	−0.007	0.502	0.035
Genetic_Env_components 2	5000	5000	5000	100	1000	SNPs across the genome	1000	10	100	0.498	−0.050	0.550	−0.002
Genetic_Env_components 3	5000	5000	5000	100	1000	SNPs across the genome	1000	20	100	0.496	−0.103	0.601	−0.044
Genetic_Env_components 4	5000	5000	5000	100	1000	SNPs across the genome	1000	30	100	0.505	−0.150	0.649	−0.079
Genetic_Env_components 5	5000	5000	5000	100	1000	SNPs across the genome	1000	40	100	0.493	−0.202	0.700	−0.128
Genetic_Env_components 6	5000	5000	5000	100	1000	SNPs across the genome	1000	50	100	0.502	−0.250	0.752	−0.166
Genetic_Env_components 7	5000	5000	5000	100	1000	SNPs across the genome	1000	60	100	0.497	−0.302	0.800	−0.211
Genetic_Env_components 8	5000	5000	5000	100	1000	SNPs across the genome	1000	70	100	0.508	−0.347	0.850	−0.246
Genetic_Env_components 9	5000	5000	5000	100	1000	SNPs across the genome	1000	80	100	0.504	−0.401	0.900	−0.288
Genetic_Env_components 10	5000	5000	5000	100	1000	SNPs across the genome	1000	90	100	0.498	−0.449	0.950	−0.330
Genetic_Env_components 11	5000	5000	5000	100	1000	SNPs across the genome	1000	100	100	0.509	−0.496	1.000	−0.373

In this simulation, we compared the agreements of the observational (calculated from phenotypes) and estimated phenotypic correlation (estimated using metaCCA) of two human traits in two samples A and B. We explored the influence of the genetic and environmental components on phenotypic correlation. More details of the simulation can be found in the Methods section. Abbreviations: N_ind_A and N_ind_B: number of individual in samples A and B; N_overlap: number of overlapped samples in samples A and B; overlap_%; percentage of overlapped samples in A and B; N_SNPs: number of SNPs in GWAS of samples A and B; SNP_region: simulated SNPs are from either one or a few LD blocks or from the whole genome; Genetic%: percentage of genetic influences on the phenotypic correlation; N_EnvF: number of environmental factors included in the model; N_simu: number of simulations; Obs_rp: observed phenotypic correlation between two traits in the mixed samples; Est_rp: mean value of the estimated phenotypic correlations in 100 simulations using metaCCA. rG and rE the simulated genetic and environmental correlation in each case.

**Table 2:** The influence of genetic and environmental components, number of SNPs, sample sizes of two GWASs, and sample overlap between two GWASs on phenotypic correlation estimation using LD score regression

Model	N_ind_A	N_ind_B	N_overlap	Overlap_%	N_SNPs	SNP_region	N_EnvF	Genetic%	N_simu	Obs_rp	Est_rp	Deviation, %
Genetic_Env_components 1	5000	5000	5000	100	200K	SNPs across the genome	1000	0	100	0.49	0.32	35.70
Genetic_Env_components 2	5000	5000	5000	100	200K	SNPs across the genome	1000	10	100	0.50	0.34	31.30
Genetic_Env_components 3	5000	5000	5000	100	200K	SNPs across the genome	1000	20	100	0.50	0.37	24.90
Genetic_Env_components 4	5000	5000	5000	100	200K	SNPs across the genome	1000	30	100	0.50	0.39	21.30
Genetic_Env_components 5	5000	5000	5000	100	200K	SNPs across the genome	1000	40	100	0.51	0.41	18.70
Genetic_Env_components 6	5000	5000	5000	100	200K	SNPs across the genome	1000	50	100	0.50	0.42	15.30
Genetic_Env_components 7	5000	5000	5000	100	200K	SNPs across the genome	1000	60	100	0.49	0.43	13.10
Genetic_Env_components 8	5000	5000	5000	100	200K	SNPs across the genome	1000	70	100	0.49	0.44	11.60
Genetic_Env_components 9	5000	5000	5000	100	200K	SNPs across the genome	1000	80	100	0.50	0.45	9.70
Genetic_Env_components 10	5000	5000	5000	100	200K	SNPs across the genome	1000	90	100	0.50	0.46	7.90
Genetic_Env_components 11	5000	5000	5000	100	200K	SNPs across the genome	1000	100	100	0.50	0.47	5.90
sample size 1	1000	1000	500	50	200K	SNPs across the genome	1000	50	100	0.50	0.22	55.10
sample size 2	3000	3000	1500	50	200K	SNPs across the genome	1000	50	100	0.51	0.30	41.70
sample size 3	5000	5000	2500	50	200K	SNPs across the genome	1000	50	100	0.50	0.33	33.30
sample size 4	10 000	10 000	5000	50	200K	SNPs across the genome	1000	50	100	0.50	0.35	30.60
sample size 5	50 000	50 000	25 000	50	200K	SNPs across the genome	1000	50	100	0.50	0.36	28.20
sample size 6	100 000	100 000	50 000	50	200K	SNPs across the genome	1000	50	100	0.51	0.39	23.90
sample overlap 1	5000	5000	500	10	200K	SNPs across the genome	1000	50	100	0.50	0.08	83.30
sample overlap 2	5000	5000	1000	20	200K	SNPs across the genome	1000	50	100	0.50	0.16	68.10
sample overlap 3	5000	5000	1500	30	200K	SNPs across the genome	1000	50	100	0.51	0.23	55.40
sample overlap 4	5000	5000	2000	40	200K	SNPs across the genome	1000	50	100	0.51	0.29	42.90
sample overlap 5	5000	5000	2500	50	200K	SNPs across the genome	1000	50	100	0.51	0.34	34.30
sample overlap 6	5000	5000	3000	60	200K	SNPs across the genome	1000	50	100	0.51	0.38	24.90
sample overlap 7	5000	5000	3500	70	200K	SNPs across the genome	1000	50	100	0.50	0.41	18.50
sample overlap 8	5000	5000	4000	80	200K	SNPs across the genome	1000	50	100	0.50	0.45	10.70
sample overlap 9	5000	5000	4500	90	200K	SNPs across the genome	1000	50	100	0.51	0.48	6.10
unbalance sample 1	5000	5000	9000	90	200K	SNPs across the genome	1000	50	100	0.50	0.47	5.90
unbalance sample 2	5000	6000	9000	82	200K	SNPs across the genome	1000	50	100	0.50	0.45	10.50
unbalance sample 3	5000	8000	9000	69	200K	SNPs across the genome	1000	50	100	0.50	0.41	18.20
unbalance sample 4	5000	10 000	9000	60	200K	SNPs across the genome	1000	50	100	0.50	0.38	23.40
unbalance sample 5	5000	13 000	9000	50	200K	SNPs across the genome	1000	50	100	0.50	0.34	31.40
number of SNPs 1	5000	5000	2500	50	7.5K	SNPs across the genome	1000	50	100	0.50	0.04	92.30
number of SNPs 2	5000	5000	2500	50	12.5K	SNPs across the genome	1000	50	100	0.50	0.11	78.20
number of SNPs 3	5000	5000	2500	50	25K	SNPs across the genome	1000	50	100	0.50	0.14	72.10
number of SNPs 4	5000	5000	2500	50	50K	SNPs across the genome	1000	50	100	0.51	0.22	56.70
number of SNPs 5	5000	5000	2500	50	100K	SNPs across the genome	1000	50	100	0.50	0.30	40.90
number of SNPs 6	5000	5000	2500	50	200K	SNPs across the genome	1000	50	100	0.51	0.34	33.70
Linkage disequilibrium 1	5000	5000	2500	50	10K	SNPs from one LD block	1000	50	100	0.51	0.09	82.30
Linkage disequilibrium 2	5000	5000	2500	50	20K	SNPs from two LD blocks	1000	50	100	0.50	0.12	75.40
Linkage disequilibrium 3	5000	5000	2500	50	30K	SNPs from three LD blocks	1000	50	100	0.50	0.16	68.80
Linkage disequilibrium 4	5000	5000	2500	50	40K	SNPs from four LD blocks	1000	50	100	0.51	0.20	60.30
Linkage disequilibrium 5	5000	5000	2500	50	50K	SNPs from five LD blocks	1000	50	100	0.50	0.22	55.70
Linkage disequilibrium 6	5000	5000	2500	50	200K	SNPs across the genome	1000	50	100	0.51	0.34	33.90

In this simulation, we compared the agreements of the observational (calculated from phenotypes) and estimated phenotypic correlation (estimated using LD score regression) of two human traits in two samples A and B. We explored the influence of the following properties: genetic and environmental components; sample size; sample overlap; unbalanced sample size in samples A and B; number of SNPs; and linkage disequilibrium. More details of the simulation can be found in the Methods section. Abbreviations: N_ind_A and N_ind_B: number of individual in samples A and B; N_overlap: number of overlapped samples in samples A and B; overlap_%: percentage of overlapped samples in A and B; N_SNPs: number of SNPs in GWAS of samples A and B; SNP_region: simulated SNPs are from either one or a few LD blocks or from the whole genome; Genetic%: percentage of genetic influences on the phenotypic correlation; N_EnvF: number of environmental factors included in the model; N_simu: number of simulations; Obs_rp: observed phenotypic correlation between two traits in the mixed samples; Est_rp: mean value of the estimated phenotypic correlations in 100 simulations; Deviation (%): deviation between observational phenotypic correlation and estimated phenotypic correlation in each model of simulation.

One important question here is how the genetic and environmental factors affect the phenotypic correlation estimation. As shown in Table 1, we found that when the environmental components dominate the phenotypic correlation, the metaCCA estimates will bias toward the null. In addition, when the genetic component dominates, metaCCA estimates will bias toward the genetic correlation. This fits the assumption that the genetic covariance matrix (built from the beta coefficients from two GWASs) will only capture the genetic effects. MetaCCA used the beta coefficients to estimate the correlation, which approximately estimates the genetic correlation within the overlapped samples. This is consistent with the simulation results in Table 1.

In contrast to metaCCA, LD score regression estimates both the genetic covariance matrix and the nongenetic covariance matrix (the error variance in the estimates of effects). In other words, given a bivariate setting (two GWASs), the slope of the LD score regression represents the genetic correlation, while the intercept term of the LD score regression represents the phenotypic correlation. This is consistent with the results in Table 2. We also found that the accuracy of the correlation estimation of LD score regression is mainly influenced by the proportion of overlapping individuals between two GWAS studies. For example, the deviation between observed and estimated phenotypic correlation improved from 83.3% to 6.1% when the percentage of sample overlap between two samples increased from 10% to 90% (Table 2). In addition, we observed that the number of SNPs included in the model will also influence the accuracy of the phenotypic correlation estimation. We also found that if all tested SNPs were from one or a few LD blocks (in other words, in high LD with each other), the accuracy of the phenotypic correlation will decrease (Table 2). Based on these two observations, we recommend including SNPs from as many genomic regions as possible to maximize the accuracy of the estimation. Finally, we observed that sample size of the GWAS influences the accuracy of the estimation, so we included GWASs with sample sizes of more than 5000 (Table 2).

**Figure 3:**
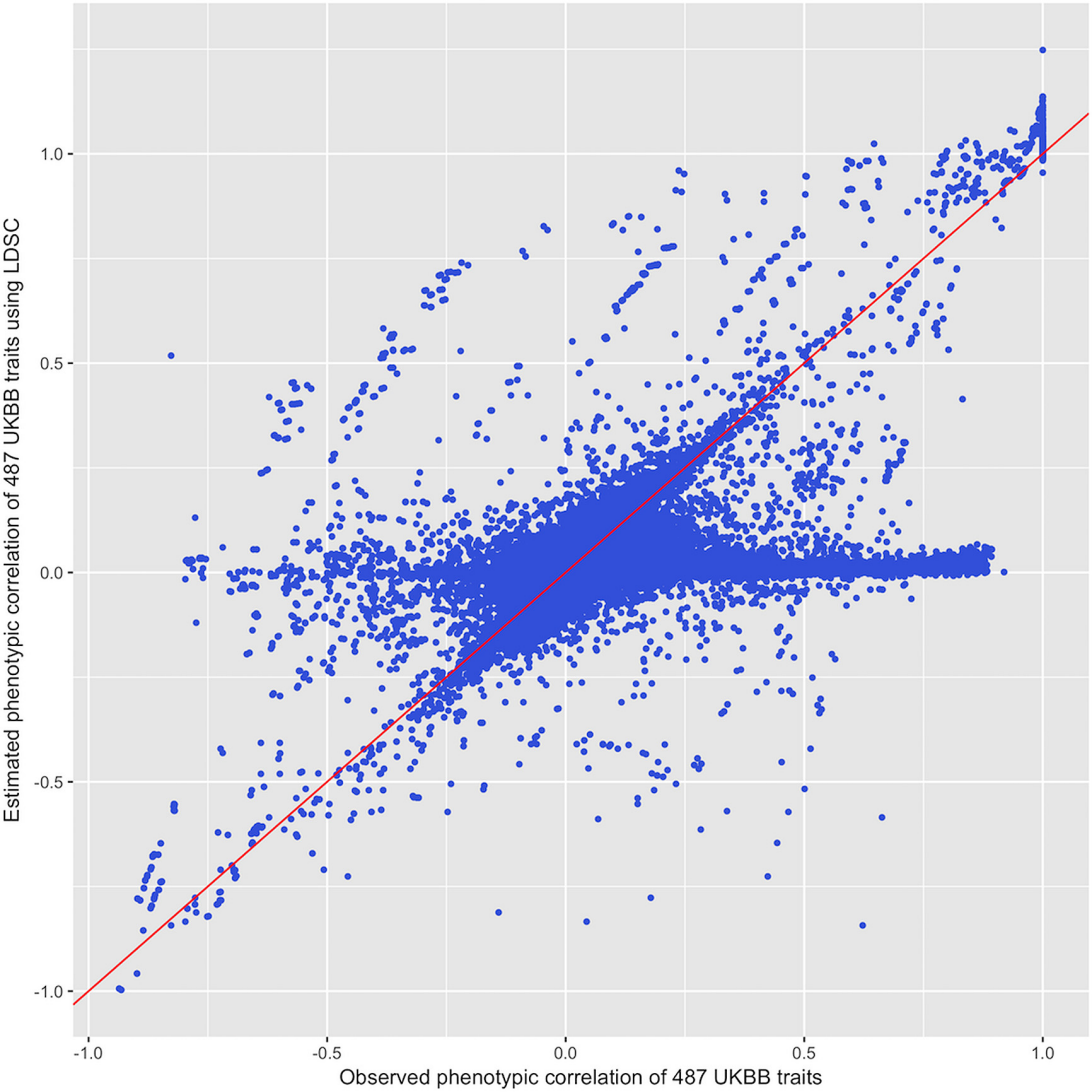
The comparison between the observed and estimated phenotypic correlations using LD score regression among 487 traits from UK Biobank. Each point is one trait. The red line is X = Y. Some traits got estimated phenotypic correlation out of bound (correlation more than one). This can occur due to the noises within the error covariance matrix (built up by the error term of the genetic association test) of a pair of traits.

We further tested the accuracy of phenotypic correlation estimation by comparing the observed phenotypic correlations (Supplementary Table S4) using real phenotype data from UK Biobank [8] and the estimated phenotypic correlation (Supplementary Table S5) using UK Biobank GWAS results via LD score regression. Fig. 3 shows that the estimated phenotypic correlations using LD score regression are consistent with the observed phenotypic correlations (r^2^ = 0.71). The exception is that some traits with large observed correlation have estimated correlation toward the null. Two possible interpretations of this discrepancy are that the phenotypic correlations of some UK Biobank traits were poorly estimated and potentially mis-specified due to limited sample size or that due to missingness of the phenotype measurements, the sample overlap was limited between some UK Biobank traits.

**Figure 4:**
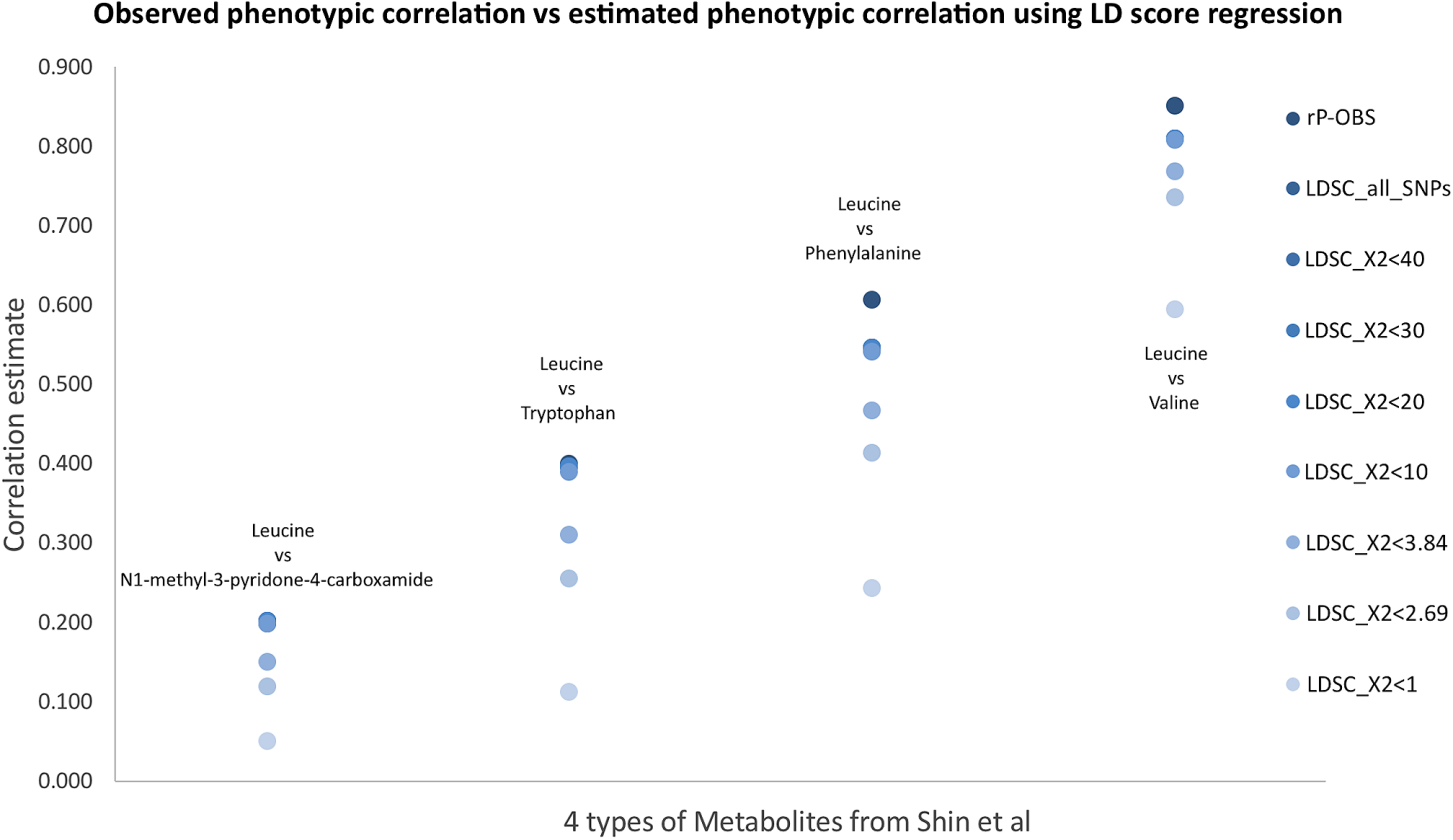
Validation of the influence of number of causal variants on phenotypic correlation estimation. Four pairs of metabolites (leucine against N1-methyl-3-pyridone-4-carboxamide, tryptophan, phenylalanine, and valine) from Shin et al. [13] were selected based on their observed phenotypic correlations (0.2, 0.4, 0.6, and 0.85, respectively). Eight sets of SNPs were selected to estimate the phenotypic correlations using LD score regression. The 8 sets were all GWAS SNPs; SNPs with Chi square statistics (square of Z scores) smaller than 40; SNPs with X^2^ < 30; SNPs with X^2^ < 20; SNPs with X^2^ < 10; SNPs with X^2^ < 3.84; SNPs with X^2^ < 2.69; and SNPs with X^2^ <1. Notes: Four columns on the *x*-axis were the four selected pairs of metabolites. The *y*-axis was the value of the phenotypic correlation. Dark blue points are the observed phenotypic correlations (noted as rP-OBS). The lighter blue points are the eight groups of SNPs included in the phenotypic correlation estimation using LD score regression (noted as LDSC_X2).

Fig. 4 illustrates the influence of the number of causal variants (which are tagged by the genetic association signals) on the accuracy of the phenotypic correlation using four pairs of metabolites from Shin et al. [13]. There is a clear trend that the estimated phenotypic correlations were further away from the observed phenotypic correlation when more and more variants with real effects were removed from the model. Based on this real case study, we recommend including all SNPs from the GWAS when estimating phenotypic correlation using LD score regression.

### A practical comparison between metaCCA and LD score regression on estimating phenotypic correlation

Both LD score regression and metaCCA have advantages and limitations when used to estimate phenotypic correlation. In this section, we summarize the practical difference between the two to inform PhenoSpD users on how to choose the appropriate methods.

LD score regression is designed to estimate genetic correlation (the slope of the regression model) between a pair of human traits. As a by-product, it also provides the pairwise phenotypic correlation estimation (the intercept of the regression model) with standard errors. It is influenced by sample overlap (when there is no sample overlap between two GWASs, the phenotypic correlation estimation will be zero). However, its application is limited to traits with large sample size (e.g., *N* >5,000), good SNP coverage (e.g., number of SNPs  >200,000), and heritable (Z score of SNP heritability >2) to fit the assumptions of LD score regression [11].

MetaCCA can be applied to almost all GWASs (e.g., in our simulation, the sample size >300 and the number of SNPs >1,000). However, it provides the approximate genetic correlation rather than the phenotypic correlation. We consider it can only be applied to the situation in which phenotypic and genetic correlation line up very well, such as metabolites [12]. It only provides the central estimation of the phenotypic correlation but no standard error and *P* value of the correlation. In addition, the method does not adjust the influence of sample overlap; to maximize the accuracy of the phenotypic correlation estimation, we could put GWASs with good sample overlap into a group and only apply metaCCA to each group of GWASs (rather than cross groups).

### The phenotypic correlations of the human metabolome

In a real case study, we applied LD score regression to the human metabolome. We estimated 5,618 pair-wise phenotypic correlations among these 107 metabolites from Kettunen et al. [10] More details of the metabolites are listed in Supplementary Table S3. The phenotypic correlations among 107 metabolites and 487 UK Biobank traits estimated by LD score regression are presented in Supplementary Tables S5 and S6.

### Multiple testing correction of the human phenome

Table 3 shows the number of independent traits for two high-dimensional, complex human traits datasets. PhenoSpD using GWAS results suggested 399.6 independent tests among 487 traits from UK Biobank, which is close to 352.4 independent tests estimated using real phenotypic correlation. For metabolites from Kettunen et al., PhenoSpD suggested 33.5 as the number of independent tests for theses metabolites, which greatly reduced the dimensionality for these complex molecular traits.

**Table 3:** Summary of number of independent traits for the complex human trait networks

First author	Category	N__traits_	N__SNPs_	N__indep_
Kettunen et al.	Metabolites	107	9 826 292	33.5
UK Biobank	All traits	487	10 879 180	399.6

Abbreviations: N__traits_: number of traits in each molecular network; N__SNPs_: number of SNPs in each network; and N__indep_: number of independent tests in each network.

## Discussion

In this study, we present an integrative method, PhenoSpD, that allows phenotypic correlation estimation and multiple testing correction for human phenome using GWAS summary statistics. We illustrate the application of PhenoSpD by estimating the phenotypic correlation structure and number of independent tests of 107 metabolites from Kettunen's study [10] and 487 UK Biobank traits for the very first time. These results showcase the ability of PhenoSpD to estimate an appropriate phenotypic correlation and multiple testing correction for complex and molecular traits when samples overlap between the GWASs.

### Advantages and limitations of PhenoSpD

There are a few key advantages of PhenoSpD. First, our proposed approach utilizes the by-products of two established methods—metaCCA and bivariate LD score regression. We extended the simulations and real-world application of the by-products of these two methods and established that metaCCA can only be applied to metabolites and that bivariate LD score regression can only be used to estimate phenotypic correlation under certain conditions (Supplementary Table S1), which adds significant value to the previous findings ( [2, 3]).

In addition, we provided a simple and user-friendly tool to correct for multiple testing for large-scale “omics” data analyses and phenome-wide association studies (PheWAS). The multiple testing correction will still be stringent (since limited sample overlap between two GWASs will drive phenotypic correlation toward null) but less stringent than Bonferroni correction. This approach is therefore particularly valuable for GWAS of complex human traits such as metabolites and large-scale biobanks. As exemplars, we cleaned and reformatted more than 594 GWAS traits and precalculated the phenotypic correlation matrix for these traits from a large-scale “omics” study and UK Biobank [10, 8]. In the GitHub repository, we also provide the precalculated phenotypic correlation matrix of 221 × 221 complex human traits in LD Hub. This greatly simplifies the process of multiple testing estimation for these traits.

Following is a description of some limitations of PhenoSpD, which are general limitations when estimating phenotypic correlation using GWAS summary statistics:

- One sample setting: The samples of the two GWASs must be from substantially overlapping samples to effectively estimate phenotypic correlation.
- Genetic or environmental components:
  - For metaCCA, when genetic components appear to dominate the phenotypic correlation, using beta coefficients to estimate phenotypic correlation will bias the estimation toward the genetic correlation. When the environmental components (“environment” here can be either shared environmental contributions or stochastic phenotypic variation [13]) dominate the phenotypic correlation, using beta coefficients to estimate phenotypic correlation will bias estimates toward the null. We consider it can only be applied to the situation in which phenotypic and genetic correlation line up very well, for example, metabolites [12].
  - For LD score regression, the method is able to capture both genetic correlation (which is represented by the slope of the regression model) and phenotypic correlation (which is represented by the intercept of the regression model). When environmental factors dominate the phenotypic correlation (which means the slope of LD score regression is close to zero), the intercept (which is built up using the error term of the SNP-trait association model) can still reconstruct a substantial component of the phenotypic correlation.
- Sample size of GWASs: We recommend sample size >5,000 for LD score regression and >300 for metaCCA.
- Number of SNPs: The number of SNPs included in the model should be more than 200,000 to get a more accurate correlation estimation.
- SNP coverage: Ideally, SNPs across the whole genome should be included in the model.

### Potential application of PhenoSpD

The main application of PhenoSpD is to determine the appropriate multiple testing correction for high-dimensional phenotypic data from a single cohort or study (e.g., metabolomics [9], epigenetics [14], transcriptomics [15], and proteomics [16] platforms that assay hundreds to thousands of traits). This approach is less stringent than the very conservative Bonferroni correction, which is inappropriate given that many phenotypes are correlated and not actually independent. In an ideal world, if the individual-level data for such studies would be easily and readily available, it would be straightforward to determine the phenotypic correlations by using-individual-level phenotype data. However, individual-level phenotype data is not as readily available as GWAS summary statistics (which are increasingly openly accessible and downloadable).

Large-scale biobanks, such as UK Biobank [8], are increasingly measuring a large number of phenotypes in the same sample. It is therefore likely to become more common for large-scale GWAS studies of diverse phenotypes to be published from the same set of participants, in contrast to the current situation of lots of GWAS from different samples with different phenotypic measurements. The proposed method will be particularly applicable for these biobanks. For example, recently automated GWAS of more than 2,400 human traits has been performed in the UK Biobank, enabling PhenoSpD analysis on a very large number of individuals (data can be downloaded from [17]).

Moreover, PheWAS is becoming a very popular tool and the dimensionality of PheWAS will increase greatly in coming years. We are moving away from single, hypothesis-driven analyses to high dimensional hypothesis-free PheWAS analyses. Tools such as PhenoSpD are therefore potentially extremely useful for PheWAS approaches such as MR-PheWAS [18] and MR-Base [4]. To maximize the value of overlapping samples in published GWAS, we recommended a specific strategy when applying PhenoSpD. The strategy is, correlated traits tend to be measured and studied within the same pool of individuals from a specific consortium. For example, anthropometric traits are mostly meta-analyzed by the GIANT consortium [19-21]; and most of the glucose- and insulin-related traits are studied in the MAGIC consortium [22-24]. We could estimate the phenotypic correlations inside each consortium. In such a way, we will be able to utilize the overlapping samples to reconstruct part of the phenotypic correlation.

In general, with the development of resources such as LD Hub and MR-Base and large-scale phenotyping and GWAS in major biobanks (e.g., UK Biobank), the proposed method, PhenoSpD, will become more relevant.

## Supplementary Material

Response_to_Reviewer_Comments_Original_Submission.pdf

Reviewer_1_Report_(Original_Submission) -- Donghyung Lee 8/29/2017 Reviewed

Reviewer_1_Report_Revision1 -- Donghyung Lee 5/21/2018 Reviewed

Reviewer_2_Report_(Original_Submission) -- Aaron Day-Williams 9/8/2017 Reviewed

Reviewer_2_Report_Revision_1 -- Aaron Day-Williams 6/7/2018 Reviewed

Supplemental Files

## Availability of supporting data

Project name: PhenoSpD

Project home page: https://github.com/MRCIEU/PhenoSpD

License: PhenoSpD is licensed under GNU GPL v3.

All data used in this manuscript are publicly available and can be downloaded from the following links:

GWAS results from Shin et al.: http://mips.helmholtz-muenchen.de/proj/GWAS/gwas/gwas_server/shin_et_al.metal.out.tar.gz

GWAS results from Kettunen et al.: http://www.computationalmedicine.fi/data#NMR_GWAS

UK Biobank GWAS results from Neale Lab: http://www.nealelab.is/blog/2017/7/19/rapid-gwas-of-thousands-of-phenotypes-for-337000-samples-in-the-uk-biobank.

Operating systems: Linux, OS X, Windows

Programming languages: R; R code and documentation for PhenoSpD V1.0.0 is available online https://github.com/MRCIEU/PhenoSpD

Research resource identifier: PhenoSpD, RRID:SCR_016359

An archival copy of the GitHub repository is also available via the *GigaScience* database, GigaDB [25].

## Abbreviations

GWAS: genome-wide association study; LD: linkage disequilibrium; PheWAS: phenome-wide association study; SNP: single nucleotide polymorphism; SpD: spectral decomposition of matrices.
