# Supplementary material for: PhenoSpD: an integrated toolkit for phenotypic correlation estimation and multiple testing correction using GWAS summary statistics
Source: Gigascience. 2018 Aug 24;7(8):giy090. doi: 10.1093/gigascience/giy090 (PMC6109640; doi:10.1093/gigascience/giy090)
Supplement: Supplemental Files [file giy090_supplemental_files.zip › PhenoSpD_maintext_revision-final-track-records.docx]

PhenoSpD: an integrated toolkit for phenotypic correlation estimation and multiple testing correction using GWAS summary statistics

Jie Zheng^1,*^, Tom G. Richardson^1^, Louise A. C. Millard^1,2^, Gibran Hemani^1^, Benjamin L. Elsworth^1^, Christopher A. Raistrick^1^, Bjarni Vilhjalmsson^3^, Benjamin M. Neale^4,5^, Philip C. Haycock^1^, George Davey Smith^1^, Tom R. Gaunt^1,*^

^1^MRC Integrative Epidemiology Unit, University of Bristol, Oakfield House, Bristol, UK; ^2^Intelligent Systems Laboratory, University of Bristol, Bristol, UK; ^3^Århus Center for Bioinformatics BIRC, Aarhus University; ^4^Program in Medical and Population Genetics, Broad Institute of MIT and Harvard, Cambridge, MA, USA; ^5^Analytical and Translational Genetics Unit, Department of Medicine, Massachusetts General Hospital and Harvard Medical School, Boston, MA, USA

*To whom correspondence should be addressed.

**Contact:** [jie.zheng@bristol.ac.uk](mailto:jie.zheng@bristol.ac.uk),

# Abstract

**Background:** Identifying phenotypic correlations between complex traits and diseases can provide useful etiological insights. Restricted access to much individual-level phenotype data makes it difficult to estimate large-scale phenotypic correlation across the human phenome. Two state-of-the-art methods, metaCCA and LD score regression, provide an alternative approach to estimate phenotypic correlation using only genome-wide association study (GWAS) summary results.

**Results:** Here, we present an integrated R toolkit, PhenoSpD, to 1) use LD score regression to estimate phenotypic correlations using GWAS summary statistics; and 2) utilize the estimated phenotypic correlations to inform correction of multiple testing for complex human traits using the spectral decomposition of matrices (SpD). The simulations suggest 1) it is possible to identify non-independence of phenotypes using samples with partial overlap, as overlap decreases the estimated phenotypic correlations will attenuate towards zero and multiple testing correction will be more stringent than in perfectly overlapping samples; 2) in contrast to LD score regression, metaCCA will provide approximate genetic correlations rather than phenotypic correlation, which limits its application for multiple testing correction. In a case study, PhenoSpD using UK Biobank GWAS results suggested 399.6 independent tests among 487 human traits, which is close to the 352.4 independent tests estimated using true phenotypic correlation. We further applied PhenoSpD to an estimated 5618 pair-wise phenotypic correlations among 107 metabolites using GWAS summary statistics from Kettunen *et al*. and PhenoSpD suggested the equivalent of 33.5 independent tests for theses metabolites.

**Conclusion:** PhenoSpD extends the use of summary level results, providing a simple and conservative way to reduce dimensionality for complex human traits using GWAS summary statistics. This is particularly valuable in the age of large-scale biobank and consortia studies, where GWAS results are much more accessible than individual-level data.

**Availability:** R code and documentation for PhenoSpD V1.0.0 is available online https://github.com/MRCIEU/PhenoSpD.

# Introduction

# Phenotypic correlations between complex human traits and diseases can provide useful etiological insights to understand mechanisms across the human phenome. However, a lack of individual-level phenotype data makes it difficult to estimate the phenotypic correlations between human traits and diseases. Fortunately, we are now in the post genome-wide association study (GWAS) era, in which many GWAS summary results are openly accessible for a large number of human diseases and traits (Pasaniuc and Price, 2017). It can therefore be valuable to use these genetic association summary statistics to reconstruct total phenotypic correlations across the human phenome. The key assumptions here are: 1) phenotypic correlation comprises both genetic and non-genetic (environmental) components; 2) the genetic association information is able to capture both genetic and non-genetic components of the phenotypic correlation (Bulik-Sullivan *et al*., 2015b).

# In this paper, we consider two methods that can be used (but were not designed) to estimate phenotypic correlations using GWAS summary statistics as by-products of the main purposes of those methods. First, MetaCCA (Cichonska *et al*., 2016) is a multivariate meta-analysis tool that allows multivariate representation of both genotype and phenotype. As a by-product, metaCCA estimates the phenotypic correlation between two traits based on a Pearson correlation between two univariable regression coefficients (betas) across a set of genetic variants. Second, bivariate linkage disequilibrium (LD) score regression (Bulik-Sullivan *et al*., 2015b) is a state-of-the-art approach to estimate genetic correlations between a pair of traits. As a consequence, the bivariate LD score regression approach allows estimation of phenotypic correlation amongst the overlapping samples of two GWASs. Assuming the genetic and non-genetic components of two phenotypes are independent, the genetic covariance matrix (built up by the beta coefficients of the genetic association test) will capture the genetic effects, while the error covariance matrix (built up by the error term of the genetic association test) will capture the environmental (non-genetic) effects. Using a bivariate LD score regression model, we are able to capture both (genetic correlation will be represented by the slope of the regression model and phenotypic correlation will be represented by the intercept of the regression model) (Bulik-Sullivan *et al*., 2015b).

# Large-scale genetic association databases such as MR-Base (Hemani *et al*., 2018) and LD Hub (Zheng *et al*., 2017) have harmonized GWAS summary-level results for roughly 1700 human traits. This provides a timely opportunity to estimate the phenotypic correlation structure across a wide range of high-dimensional, complex molecular traits, such as metabolites, that are potentially highly correlated. Bonferroni correction would markedly overcorrect for the inflated false-positive rate in such correlated datasets, resulting in a reduction in power. An appropriate method to correct for multiple testing among human traits and diseases is the spectral decomposition of matrices (SpD) (Nyholt, 2004; Li and Ji, 2005). In this manuscript we combine LD score regression with SpD to estimate number of independent tests using only summary-level GWAS data.

# Methods

**Overview of PhenoSpD**

#
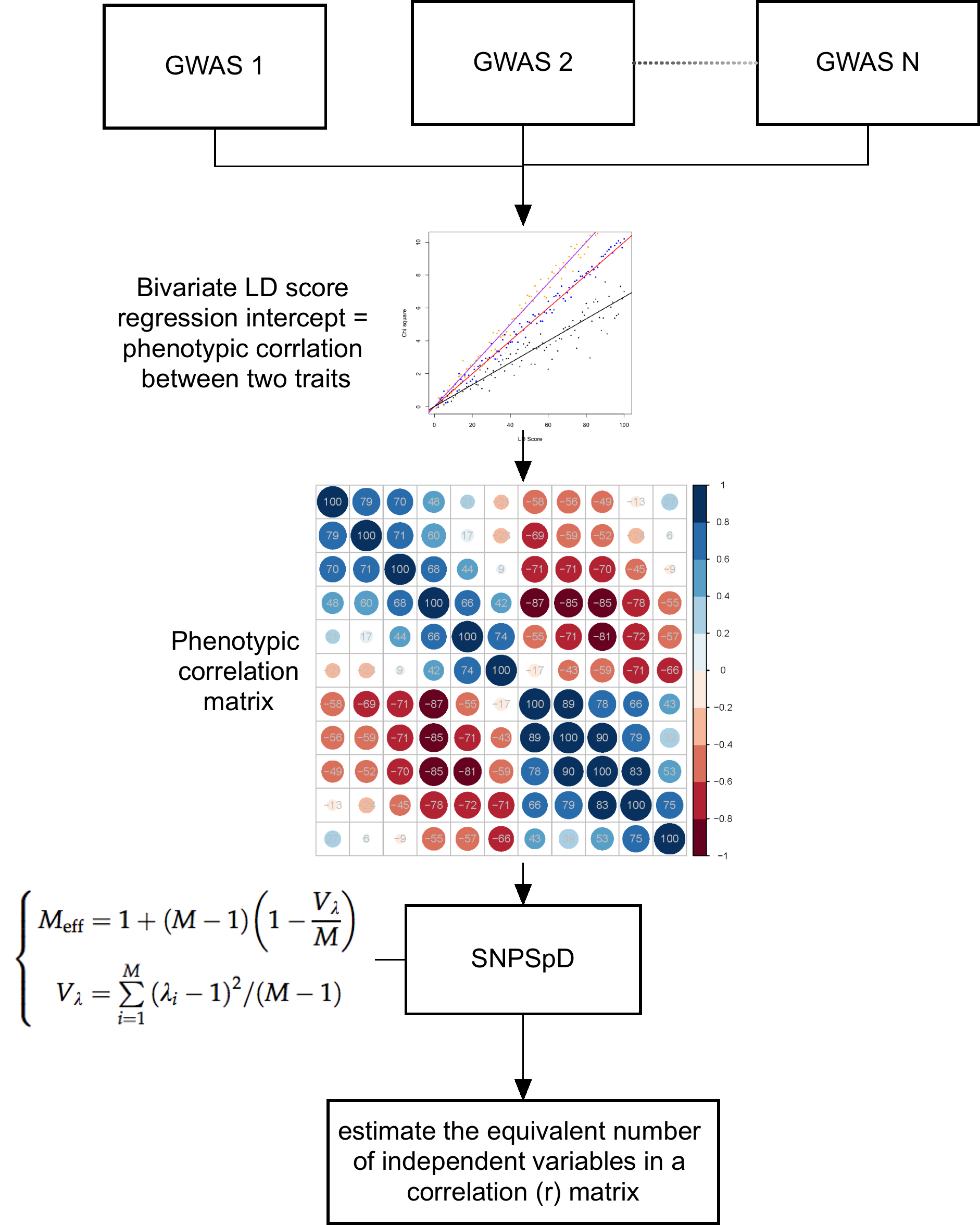
Figure 1 illustrates the key steps of the proposed pipeline, PhenoSpD: step (1) harmonise GWAS summary results from the same sample; step (2) apply the harmonized GWAS results to LD score regression to estimate the phenotypic correlation matrix of the traits; step (3) apply the SpD approach to the phenotypic correlation matrix and estimate the number of independent variables among the traits.

**Figure 1.** Flowchart of PhenoSpD.

**Simulation of phenotypic correlation estimation**

# Firstly, we simulated the influence of the number of single nucleotide polymorphisms (SNPs), sample sizes of two GWASs and sample overlap between two GWASs on the accuracy of the phenotypic correlation estimation. As shown in Figure 2, we first created two samples A and B with different number of individuals (from 300 to 10,000 individuals), where the sample overlap between sample A and B ranged from 10% to 90%. We assumed complex human traits were influenced by both genetic and environmental factors, so we simulated the phenotype data of two correlated human traits (phenotype 1 and phenotype 2 with a phenotypic correlation of -0.7) based on varying numbers of genetic factors (ranging from 10 to 10,000 SNPs), different LD structure (r^2^ between 0 to 0.9) and 100 environmental factors. We then assigned the phenotypic correlation to its genetic and environmental components, each of which explained 10% to 90% of the total phenotypic variance. These genetic and environmental components were further assigned to each of the genetic and environmental factors in the model randomly. We also simulated two extreme cases where either the genetic or environmental components dominate the phenotypic correlation. After simulating the two phenotypic traits and the genotypic data in sample A and B, we then conducted four GWASs (GWASs of phenotype 1 in sample A and B; GWASs of phenotype 2 in sample A and B) and recorded the summary statistics of these GWASs. To measure the accuracy of phenotypic correlation using GWAS summary statistics, we (1) calculated the observational phenotypic correlation (the Pearson correlation) between trait 1 and trait 2 in sample A and B separately; (2) estimated the phenotypic correlation between trait 1 and trait 2 in the overlapped samples using both metaCCA and LD score regression. We simulated step (2) 100 times and estimated the mean and standard deviation of the estimated phenotypic correlations. Finally, we compared the estimated phenotypic correlation with the observational phenotypic correlation and recorded the deviation between observed and estimated correlations. To demonstrate the simulation systematically, we explored the influence of the following properties: (i) the influence of sample size; (ii) sample overlap; (iii) unbalanced sample size in sample A and B; (iv) number of SNPs; and (v) LD. The R script for this simulation is provided as a supplementary file (simulation.R).

**
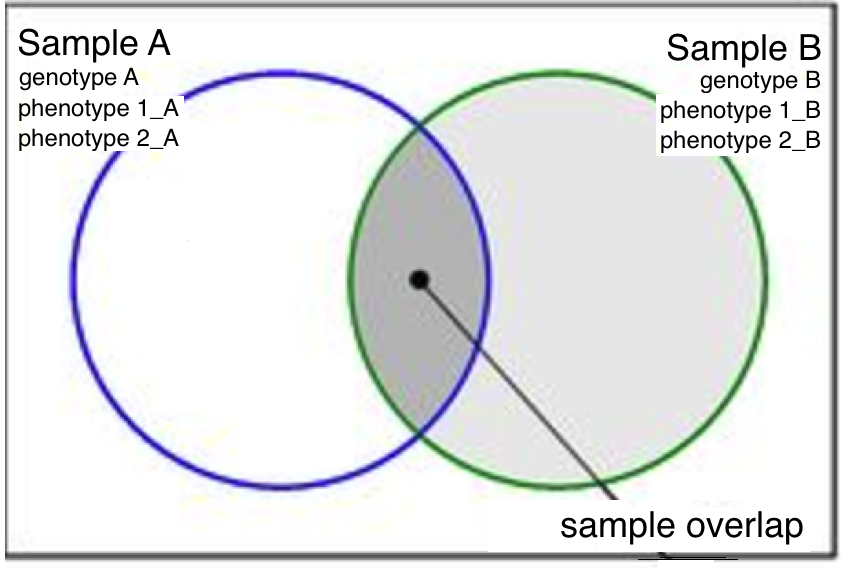
**

**Figure 2.** Demonstration of the simulation. For two samples A and B, we simulated the genotype data and phenotype data of two correlated human traits, phenotype 1 and phenotype 2. The sample overlap between sample A and B ranged from 10% to 90% in this simulation.

**Validation of phenotypic correlation estimation using real GWAS data**

# We further tested the accuracy of the phenotypic correlation estimation using GWAS summary statistics of 487 traits from the UK Biobank (Sudlow *et al.,* 2015) (Table S2). We calculated the observational phenotypic correlation using the actual phenotype data (Table S4), which was used as benchmark to evaluate the accuracy of our phenotypic correlation estimates using LD score regression.

# In addition, we tested whether the number of causal variants (which are tagged by the genetic association signals) may affect the accuracy of the phenotypic correlation using 4 pairs of metabolites from Shin *et al*. The 4 pairs of metabolites were selected because they have a wide range of observed phenotypic correlation from 0.2 to 0.85. To validate the accuracy, we compared the observed phenotypic correlation with the phenotypic correlation estimated by LD score regression. To consider number of causal variants in this validation, we setup 8 groups of SNPs based on their effects on the traits. The 8 groups were (1) all GWAS SNPs; (2) SNPs with Chi square statistics (square of Z scores) smaller than 40; (3) SNPs with X^2^ < 30; (4) SNPs with X^2^ < 20; (5) SNPs with X^2^ < 10; (6) SNPs with X^2^ < 3.84; (7) SNPs with X^2^ < 2.69 and (8) SNPs with X^2^ < 1. In other words, we progressively reduced the number of casual variants from the model and evaluated the impact of this on the accuracy of the phenotypic correlation estimation.

Based on the simulation and real case validation, we listed our traits selection criteria in Table S1.

**Estimating the phenotypic correlations**

Within our GWAS summary results database containing roughly 1700 human traits, we selected 107 metabolites from Kettunen *et al.* as a real case application (Kettunen *et al.*, 2016) since these complex molecular traits are potentially highly correlated. We then applied LD score regression to these 107 metabolites to estimate the phenotypic correlation matrix (Table S3), which meets the suggested minimum parameters of the LD score regression method (traits with large sample size (e.g. N > 5,000), good SNP coverage (e.g. number of SNPs > 200,000) and heritable (e.g. Z score of the SNP heritability > 2)).

**Multiple testing correction for human traits**

We applied the SpD approach to estimate the number of independent tests among the 107 metabolites and 487 UK Biobank traits. The observed phenotypic correlations and correlations estimated by LD score regression were used as input for the SpD approach. We implemented the R code of the well-known method, SNPSpD (Nyholt, 2004; Li and Ji, 2005), to estimate the number of independent traits using the phenotypic correlation matrix as input (Fig. 1). The output of the SpD function is the estimated number of independent tests.

# Results

**Evaluation of phenotypic correlation estimation using simulated and real GWAS summary data**

Tables 1 and 2 show the influence of changing various parameters on the accuracy of the phenotypic correlation estimation for metaCCA and LD score regression respectively. Our general observations from the simulation are 1) since the genetic association information is able to capture both genetic and non-genetic components of the phenotypic correlation, we can estimate such correlation for any human trait, even for non-heritable traits. 2) we should apply LD score regression to estimate phenotypic correlation in a one sample setting (i.e. where all GWAS are performed in the same sample). It is possible to identify non-independence of phenotypes using GWAS results from samples with only a partial overlap, but as overlap decreases correlations will attenuate towards zero. 3) metaCCA will provide approximate genetic correlations rather than phenotypic correlation, which limits its application in our approach to evaluating multiple testing.

One important question here is how the genetic and environmental factors affect the phenotypic correlation estimation. As shown in Table 1, we found that when the environmental components dominate the phenotypic correlation, the metaCCA estimates will bias towards the null. In addition, when the genetic component dominates, metaCCA estimates will bias towards the genetic correlation. This fits the assumption that the genetic covariance matrix (built from the beta coefficients from two GWASs) will only capture the genetic effects. MetaCCA used the beta coefficients to estimate the correlation, which approximately estimates the genetic correlation within the overlapped samples. This is consistent with the simulation results in Table 1.

In contrast to metaCCA, LD score regression estimates both the genetic covariance matrix and the non-genetic covariance matrix (the error variance in the estimates of effects). In other words, given a bivariate setting (two GWASs), the slope of the LD score regression represents the genetic correlation, while the intercept term of the LD score regression represents the phenotypic correlation. This is consistent with the results in Table 2. We also found that the accuracy of the correlation estimation of LD score regression is mainly influenced by the proportion of overlapping individuals between two GWAS studies. For example, the deviation between observed and estimated phenotypic correlation improved from 83.3% to 6.1% when the percentage of sample overlap between two samples increased from 10% to 90% (Table 2). In addition, we observed that the number of SNPs included in the model will also influence the accuracy of the phenotypic correlation estimation. We also found that if all tested SNPs were from one or few LD blocks (in other words, in high LD with each other), the accuracy of the phenotypic correlation will decrease (Table 2). Based on these two observations, we recommend including SNPs from as many genomic regions as possible to maximize the accuracy of the estimation. Finally, we observed that sample size of the GWAS influences the accuracy of the estimation, so we included GWASs with sample sizes more than 5000 (Table 2).

We further tested the accuracy of phenotypic correlation estimation by comparing the observed phenotypic correlations (Table S4) using real phenotype data from UK Biobank (Sudlow *et al.,* 2015) and the estimated phenotypic correlation (Table S5) using UK Biobank GWAS results via LD score regression. Figure 3 shows the estimated phenotypic correlations using LD score regression are consistent with the observed phenotypic correlations (r^2^ =0.71). The exception is that some traits with large observed correlation have estimated correlation towards null. Two possible interpretations of this discrepancy are (1) The phenotypic correlations of some UK Biobank traits were poorly estimated and potentially mis-specified due to limited sample size; (2) due to missingness of the phenotype measurements, the sample overlap was limited between some UK Biobank traits.

Figure 4 illustrates the influence of the number of causal variants (which are tagged by the genetic association signals) on the accuracy of the phenotypic correlation using 4 pairs of metabolites from Shin *et al*. There is a clear trend that the estimated phenotypic correlations were further away from the observed phenotypic correlation when more and more variants with real effects were removed from the model. Based on this real case study we recommend including all SNPs from the GWAS when estimating phenotypic correlation using LD score regression.

**Table 1.** The influence of genetic and environmental components on phenotypic correlation estimation using metaCCA.

| Model | N_ind_A | | N_ind_B | | N_overlap | | Overlap_% | | N_SNPs | SNP_region | N_EnvF | Genetic% | | N_simu | | Obs_rp | | rG | | rE | Est_rp | | |  | |
| --- | --- | --- | --- | --- | --- | --- | --- | --- | --- | --- | --- | --- | --- | --- | --- | --- | --- | --- | --- | --- | --- | --- | --- | --- | --- |
| Genetic_Env_components 1 | 5000 | | 5000 | | 5000 | | 100% | | 1000 | SNPs across the genome | 1000 | 0% | | 100 | | 0.497 | | -0.007 | | 0.502 | 0.035 | | |  | |
| Genetic_Env_components 2 | 5000 | | 5000 | | 5000 | | 100% | | 1000 | SNPs across the genome | 1000 | 10% | | 100 | | 0.498 | | -0.050 | | 0.550 | -0.002 | | |  | |
| Genetic_Env_components 3 | 5000 | | 5000 | | 5000 | | 100% | | 1000 | SNPs across the genome | 1000 | 20% | | 100 | | 0.496 | | -0.103 | | 0.601 | -0.044 | | |  | |
| Genetic_Env_components 4 | 5000 | | 5000 | | 5000 | | 100% | | 1000 | SNPs across the genome | 1000 | 30% | | 100 | | 0.505 | | -0.150 | | 0.649 | -0.079 | | |  | |
| Genetic_Env_components 5 | 5000 | | 5000 | | 5000 | | 100% | | 1000 | SNPs across the genome | 1000 | 40% | | 100 | | 0.493 | | -0.202 | | 0.700 | -0.128 | |  | |  |
| Genetic_Env_components 6 | 5000 | | 5000 | | 5000 | | 100% | | 1000 | SNPs across the genome | 1000 | 50% | | 100 | | 0.502 | | -0.250 | | 0.752 | -0.166 | | |  | |
| Genetic_Env_components 7 | 5000 | | 5000 | | 5000 | | 100% | | 1000 | SNPs across the genome | 1000 | 60% | | 100 | | 0.497 | | -0.302 | | 0.800 | -0.211 | | |  | |
| Genetic_Env_components 8 | 5000 | | 5000 | | 5000 | | 100% | | 1000 | SNPs across the genome | 1000 | 70% | | 100 | | 0.508 | | -0.347 | | 0.850 | -0.246 | | |  | |
| Genetic_Env_components 9 | 5000 | | 5000 | | 5000 | | 100% | | 1000 | SNPs across the genome | 1000 | 80% | | 100 | | 0.504 | | -0.401 | | 0.900 | -0.288 | | | | |
| Genetic_Env_components 10 | 5000 | | 5000 | | 5000 | | 100% | | 1000 | SNPs across the genome | 1000 | 90% | | 100 | | 0.498 | | -0.449 | | 0.950 | -0.330 | | | | |
| Genetic_Env_components 11 | 5000 | | 5000 | | 5000 | | 100% | | 1000 | SNPs across the genome | 1000 | 100% | | 100 | | 0.509 | | -0.496 | | 1.000 | -0.373 | | | | |

In this simulation, we compared the agreements of the observational (calculated from phenotypes) and estimated phenotypic correlation (estimated using metaCCA) of two human traits in two samples A and B. We explored the influence of the genetic and environmental components on phenotypic correlation. More details of the simulation can be found in methods section. Abbreviations: N_ind_A and N_ind_B, number of individual in sample A and B. N_overlap, number of overlapped samples in sample A and B. overlap_%, the percentage of over-lapped samples in A and B. N_SNPs, number of SNPs in GWAS of sample A and B. SNP_region, the simulated SNPs are from either one or few LD blocks or from the whole genome. Genetic%, the percentage of genetic influences on the phenotypic correlation; N_EnvF, number of environmental factors included in the model. N_simu, number of simulations. Obs_rp, the observed phenotypic correlation between two traits in the mixed samples. Est_rp, the mean value of the estimated phenotypic correlations in 100 simulations using metaCCA. rG and rE the simulated genetic and environmental correlation in each case.

**Table 2.** The influence of genetic and environmental components, number of SNPs, sample sizes of two GWASs and sample overlap between two GWASs on phenotypic correlation estimation using LD score regression.

| Model | N_ind_A | N_ind_B | N_overlap | Overlap_% | N_SNPs | SNP_region | N_EnvF | Genetic% | N_simu | Obs_rp | Est_rp | Deviation (%) |
| --- | --- | --- | --- | --- | --- | --- | --- | --- | --- | --- | --- | --- |
| Genetic_Env_components 1 | 5000 | 5000 | 5000 | 100% | 200K | SNPs across the genome | 1000 | 0% | 100 | 0.49 | 0.32 | 35.70% |
| Genetic_Env_components 2 | 5000 | 5000 | 5000 | 100% | 200K | SNPs across the genome | 1000 | 10% | 100 | 0.50 | 0.34 | 31.30% |
| Genetic_Env_components 3 | 5000 | 5000 | 5000 | 100% | 200K | SNPs across the genome | 1000 | 20% | 100 | 0.50 | 0.37 | 24.90% |
| Genetic_Env_components 4 | 5000 | 5000 | 5000 | 100% | 200K | SNPs across the genome | 1000 | 30% | 100 | 0.50 | 0.39 | 21.30% |
| Genetic_Env_components 5 | 5000 | 5000 | 5000 | 100% | 200K | SNPs across the genome | 1000 | 40% | 100 | 0.51 | 0.41 | 18.70% |
| Genetic_Env_components 6 | 5000 | 5000 | 5000 | 100% | 200K | SNPs across the genome | 1000 | 50% | 100 | 0.50 | 0.42 | 15.30% |
| Genetic_Env_components 7 | 5000 | 5000 | 5000 | 100% | 200K | SNPs across the genome | 1000 | 60% | 100 | 0.49 | 0.43 | 13.10% |
| Genetic_Env_components 8 | 5000 | 5000 | 5000 | 100% | 200K | SNPs across the genome | 1000 | 70% | 100 | 0.49 | 0.44 | 11.60% |
| Genetic_Env_components 9 | 5000 | 5000 | 5000 | 100% | 200K | SNPs across the genome | 1000 | 80% | 100 | 0.50 | 0.45 | 9.70% |
| Genetic_Env_components 10 | 5000 | 5000 | 5000 | 100% | 200K | SNPs across the genome | 1000 | 90% | 100 | 0.50 | 0.46 | 7.90% |
| Genetic_Env_components 11 | 5000 | 5000 | 5000 | 100% | 200K | SNPs across the genome | 1000 | 100% | 100 | 0.50 | 0.47 | 5.90% |
| sample size 1 | 1000 | 1000 | 500 | 50% | 200K | SNPs across the genome | 1000 | 50% | 100 | 0.50 | 0.22 | 55.10% |
| sample size 2 | 3000 | 3000 | 1500 | 50% | 200K | SNPs across the genome | 1000 | 50% | 100 | 0.51 | 0.30 | 41.70% |
| sample size 3 | 5000 | 5000 | 2500 | 50% | 200K | SNPs across the genome | 1000 | 50% | 100 | 0.50 | 0.33 | 33.30% |
| sample size 4 | 10000 | 10000 | 5000 | 50% | 200K | SNPs across the genome | 1000 | 50% | 100 | 0.50 | 0.35 | 30.60% |
| sample size 5 | 50000 | 50000 | 25000 | 50% | 200K | SNPs across the genome | 1000 | 50% | 100 | 0.50 | 0.36 | 28.20% |
| sample size 6 | 100000 | 100000 | 50000 | 50% | 200K | SNPs across the genome | 1000 | 50% | 100 | 0.51 | 0.39 | 23.90% |
| sample overlap 1 | 5000 | 5000 | 500 | 10% | 200K | SNPs across the genome | 1000 | 50% | 100 | 0.50 | 0.08 | 83.30% |
| sample overlap 2 | 5000 | 5000 | 1000 | 20% | 200K | SNPs across the genome | 1000 | 50% | 100 | 0.50 | 0.16 | 68.10% |
| sample overlap 3 | 5000 | 5000 | 1500 | 30% | 200K | SNPs across the genome | 1000 | 50% | 100 | 0.51 | 0.23 | 55.40% |
| sample overlap 4 | 5000 | 5000 | 2000 | 40% | 200K | SNPs across the genome | 1000 | 50% | 100 | 0.51 | 0.29 | 42.90% |
| sample overlap 5 | 5000 | 5000 | 2500 | 50% | 200K | SNPs across the genome | 1000 | 50% | 100 | 0.51 | 0.34 | 34.30% |
| sample overlap 6 | 5000 | 5000 | 3000 | 60% | 200K | SNPs across the genome | 1000 | 50% | 100 | 0.51 | 0.38 | 24.90% |
| sample overlap 7 | 5000 | 5000 | 3500 | 70% | 200K | SNPs across the genome | 1000 | 50% | 100 | 0.50 | 0.41 | 18.50% |
| sample overlap 8 | 5000 | 5000 | 4000 | 80% | 200K | SNPs across the genome | 1000 | 50% | 100 | 0.50 | 0.45 | 10.70% |
| sample overlap 9 | 5000 | 5000 | 4500 | 90% | 200K | SNPs across the genome | 1000 | 50% | 100 | 0.51 | 0.48 | 6.10% |
| unbalance sample 1 | 5000 | 5000 | 9000 | 90% | 200K | SNPs across the genome | 1000 | 50% | 100 | 0.50 | 0.47 | 5.90% |
| unbalance sample 2 | 5000 | 6000 | 9000 | 82% | 200K | SNPs across the genome | 1000 | 50% | 100 | 0.50 | 0.45 | 10.50% |
| unbalance sample 3 | 5000 | 8000 | 9000 | 69% | 200K | SNPs across the genome | 1000 | 50% | 100 | 0.50 | 0.41 | 18.20% |
| unbalance sample 4 | 5000 | 10000 | 9000 | 60% | 200K | SNPs across the genome | 1000 | 50% | 100 | 0.50 | 0.38 | 23.40% |
| unbalance sample 5 | 5000 | 13000 | 9000 | 50% | 200K | SNPs across the genome | 1000 | 50% | 100 | 0.50 | 0.34 | 31.40% |
| number of SNPs 1 | 5000 | 5000 | 2500 | 50% | 7.5K | SNPs across the genome | 1000 | 50% | 100 | 0.50 | 0.04 | 92.30% |
| number of SNPs 2 | 5000 | 5000 | 2500 | 50% | 12.5K | SNPs across the genome | 1000 | 50% | 100 | 0.50 | 0.11 | 78.20% |
| number of SNPs 3 | 5000 | 5000 | 2500 | 50% | 25K | SNPs across the genome | 1000 | 50% | 100 | 0.50 | 0.14 | 72.10% |
| number of SNPs 4 | 5000 | 5000 | 2500 | 50% | 50K | SNPs across the genome | 1000 | 50% | 100 | 0.51 | 0.22 | 56.70% |
| number of SNPs 5 | 5000 | 5000 | 2500 | 50% | 100K | SNPs across the genome | 1000 | 50% | 100 | 0.50 | 0.30 | 40.90% |
| number of SNPs 6 | 5000 | 5000 | 2500 | 50% | 200K | SNPs across the genome | 1000 | 50% | 100 | 0.51 | 0.34 | 33.70% |
| Linkage disequilibrium 1 | 5000 | 5000 | 2500 | 50% | 10K | SNPs from one LD block | 1000 | 50% | 100 | 0.51 | 0.09 | 82.30% |
| Linkage disequilibrium 2 | 5000 | 5000 | 2500 | 50% | 20K | SNPs from two LD blocks | 1000 | 50% | 100 | 0.50 | 0.12 | 75.40% |
| Linkage disequilibrium 3 | 5000 | 5000 | 2500 | 50% | 30K | SNPs from three LD blocks | 1000 | 50% | 100 | 0.50 | 0.16 | 68.80% |
| Linkage disequilibrium 4 | 5000 | 5000 | 2500 | 50% | 40K | SNPs from four LD blocks | 1000 | 50% | 100 | 0.51 | 0.20 | 60.30% |
| Linkage disequilibrium 5 | 5000 | 5000 | 2500 | 50% | 50K | SNPs from five LD blocks | 1000 | 50% | 100 | 0.50 | 0.22 | 55.70% |
| Linkage disequilibrium 6 | 5000 | 5000 | 2500 | 50% | 200K | SNPs across the genome | 1000 | 50% | 100 | 0.51 | 0.34 | 33.90% |

In this simulation, we compared the agreements of the observational (calculated from phenotypes) and estimated phenotypic correlation (estimated using LD score regression) of two human traits in two samples A and B. We explored the influence of the following properties: (i) genetic and environmental components; (ii) sample size; (iii) sample overlap; (iv) unbalanced sample size in sample A and B; (v) number of SNPs; and (vi) linkage disequilibrium. More details of the simulation can be found in methods section. Abbre-viations: N_ind_A and N_ind_B, number of individual in sample A and B. N_overlap, number of overlapped samples in sample A and B. overlap_%, the percentage of over-lapped samples in A and B. N_SNPs, number of SNPs in GWAS of sample A and B. SNP_region, the simulated SNPs are from either one or few LD blocks or from the whole genome. Genetic%, the percentage of genetic influences on the phenotypic correlation; N_EnvF, number of environmental factors included in the model. N_simu, number of simulations. Obs_rp, the observed phenotypic correlation between two traits in the mixed samples. Est_rp, the mean value of the estimated phenotypic correlations in 100 simulations; Deviation (%), the deviation between observational phenotypic correlation and estimated phenotypic correlation in each model of simulation.


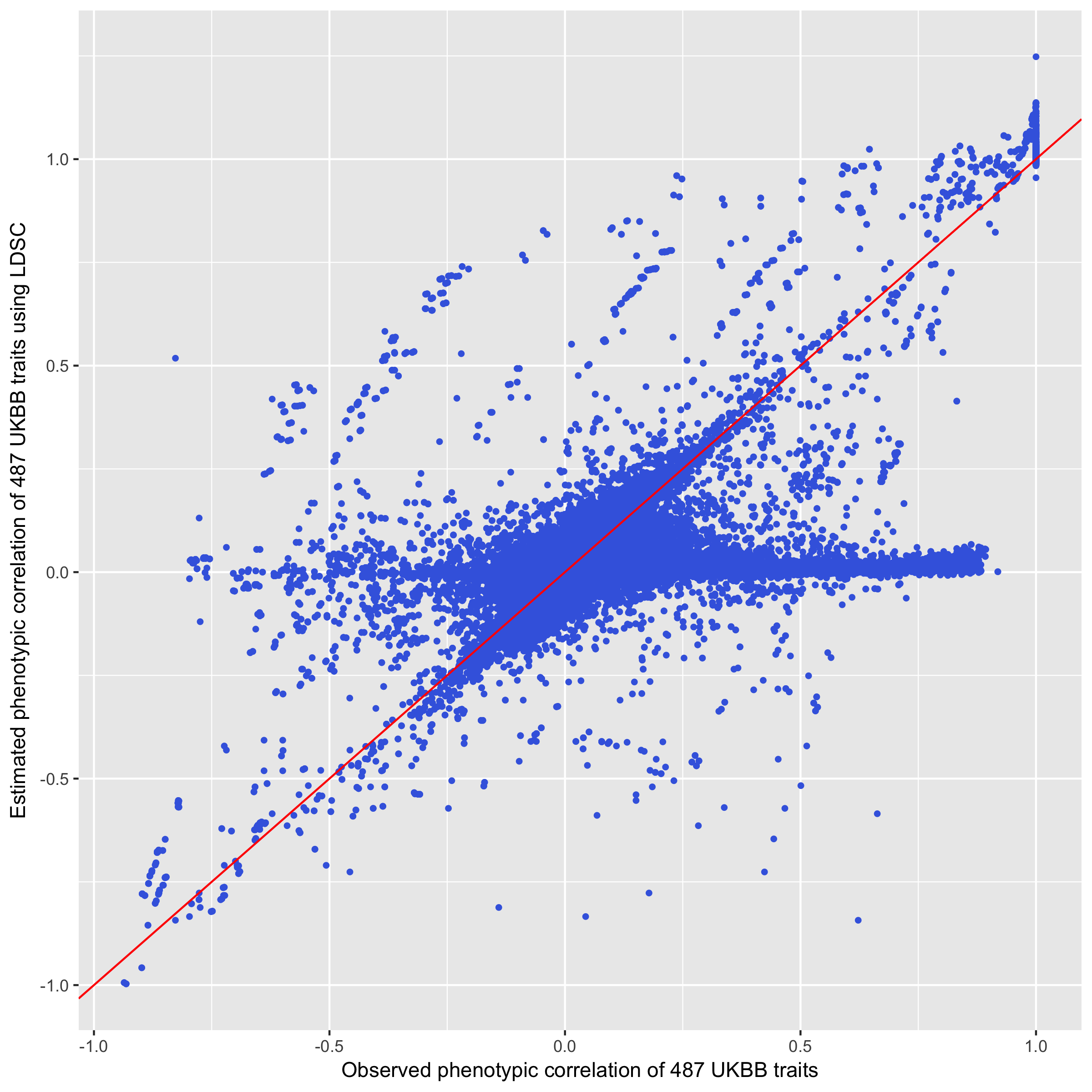


**Figure 3.** The comparison between the observed and estimated phenotypic correlations using LD score regression amongst 487 traits from UK Biobank. Each point is one trait. The red line is X=Y. Some traits got estimated phenotypic correlation slightly more than one. This can occur due to the noises within error covariance matrix (built up by the error term of the genetic association test) of a pair of traits.


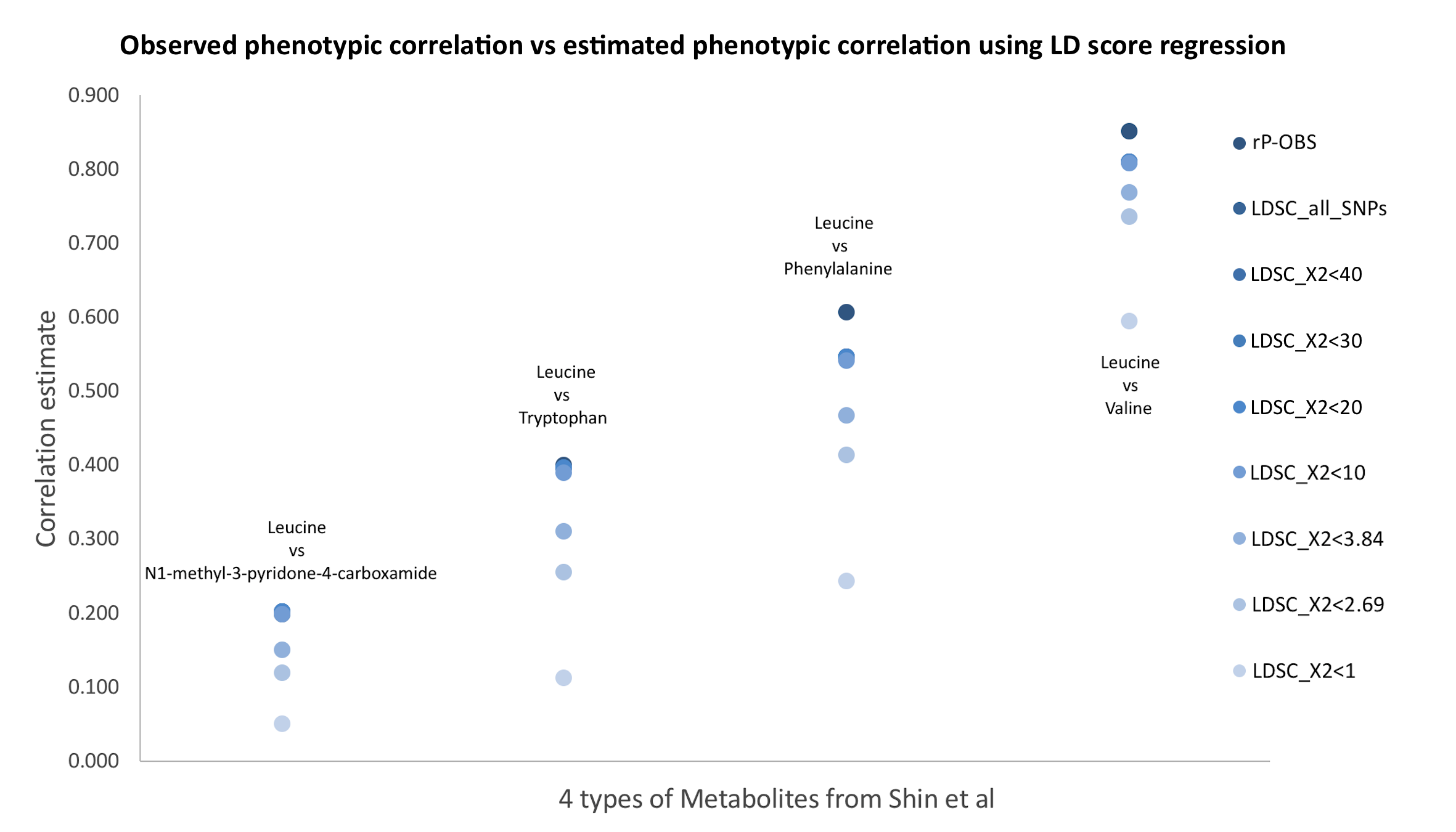


**Figure 4.** Validation of the influence of number of causal variants on phenotypic correlation estimation. 4 pairs of metabolites (leucine against N1-methyl-3-pyridone-4-carboxamide, tryptophan, phenylalanine and valine) from Shin *et al.* were selected based on their observed phenotypic correlations (0.2, 0.4, 0.6 and 0.85 respectively). 8 sets of SNPs were selected to estimate the phenotypic correlations using LD score regression. The 8 sets were (1) all GWAS SNPs; (2) SNPs with Chi square statistics (square of Z scores) smaller than 40; (3) SNPs with X^2^ < 30; (4) SNPs with X^2^ < 20; (5) SNPs with X^2^ < 10; (6) SNPs with X^2^ < 3.84; (7) SNPs with X^2^ < 2.69 and (8) SNPs with X^2^ < 1. Notes: 4 columns on the x-axis were the four selected pairs of metabolites. Y-axis was the value of the phenotypic correlation. Dark blue points are the observed phenotypic correlations (noted as rP-OBS). The points with lighter blues are the 8 groups of SNPs included in the phenotypic correlation estimation using LD score regression (noted as LDSC_X2).

**A practical comparison between metaCCA and LD score regression on estimating phenotypic correlation**

Both LD score regression and metaCCA have advantages and limitations when used to estimate phenotypic correlation. In this section, we summarise the practical difference between the two to inform PhenoSpD users on how to choose the appropriate methods.

LD score regression is designed to estimate genetic correlation (the slope of the regression model) between a pair of human traits. As a by-product, it also provides the pairwise phenotypic correlation estimation (the intercept of the regression model) with standard errors. It is influenced by sample overlap (when there is no sample overlap between two GWASs, the phenotypic correlation estimation will be zero). However, its application is limited to traits with large sample size (e.g. N > 5,000), good SNP coverage (e.g. number of SNPs > 200,000) and heritable (Z score of SNP heritability > 2) to fit the assumptions of LD score regression (Bulik-Sullivan *et al*., 2015a).

MetaCCA can be applied to almost all GWASs (e.g. in our simulation, the sample size>300 and the number of SNPs>1000). However, 1) it provides the approximate genetic correlation rather than the phenotypic correlation. We consider it can only be applied to the situation in which phenotypic and genetic correlation line up very well, such as metabolites (Würtz P, *et al*., 2014); 2) it only provides the central estimation of the phenotypic correlation but no standard error and p-value of the correlation; 2) the method does not adjust the influence of sample overlap, to maximise the accuracy of the phenotypic correlation estimation, we could put GWASs with good sample overlap into a group and only apply metaCCA to each group of GWASs (rather than cross groups).

**The phenotypic correlations of the human metabolome**

In a real case study, we applied LD score regression to the human metabolome. We estimated 5,618 pair-wise phenotypic correlations among these 107 metabolites from Kettunen *et al.* (Kettunen *et al.*, 2016) More details of the metabolites are listed in Table S3. The phenotypic correlations among 107 metabolites and 487 UK Biobank traits estimated by LD score regression are presented in Table S5 and Table S6.

**Multiple testing correction of the human phenome**

Table 3 shows the number of independent traits for two high-dimensional, complex human traits datasets. PhenoSpD using GWAS results suggested 399.6 independent tests among 487 traits from UK Biobank, which is close to 352.4 independent tests estimated using real phenotypic correlation. For metabolites from Kettunen *et al.*, PhenoSpD suggested 33.5 number of independent tests for theses metabolites, which greatly reduced the dimensionality for these complex molecular traits.

**Table 3.** Summary of number of independent traits for the complex human trait networks.

| First author | Category | N__traits_ | N__SNPs_ | N__indep_ |
| --- | --- | --- | --- | --- |
| Kettunen *et al.* | Metabolites | 107 | 9826292 | 33.5 |
| UK Biobank | All traits | 487 | 10879180 | 399.6 |

Note: N__traits_ refers to number of traits in each molecular network; N__SNPs_ refers to number of SNPs in each network; N__indep_ refers to number of independent tests in each network.

# Discussion

In this study, we present an integrative method, PhenoSpD, which allows phenotypic correlation estimation and multiple testing correction for human phenome using GWAS summary statistics. We illustrate the application of PhenoSpD by estimating the phenotypic correlation structure and number of independent tests of 107 metabolites from Kettunen’s study (Kettunen *et al.*, 2016) and 487 UK Biobank traits for the very first time. These results showcase the ability of PhenoSpD to estimate an appropriate phenotypic correlation and multiple testing correction for complex and molecular traits when samples overlap between the GWASs.

**Advantages and limitations of PhenoSpD**

There are a few key advantages of PhenoSpD. Firstly, our proposed approach utilizes the by-products of two established methods – metaCCA and bivariate LD score regression. We extended the simulations and real-world application of the by-products of these two methods and established that metaCCA can only be applied to metabolites and bivariate LD score regression can only be used to estimate phenotypic correlation under certain conditions (Table S1), which adds significant value to the previous findings (Cichonska. *et al*., 2016; Bulik-Sullivan. *et al.,* 2015).

In addition, we provided a simple and user-friendly tool to correct for multiple testing for large scale “omics” data analyses and phenome-wide association studies (PheWAS). The multiple testing correction will still be stringent (since limited sample overlap between two GWASs will drive phenotypic correlation towards null), but less stringent than Bonferroni correction. This approach is therefore particularly valuable for GWAS of complex human traits such as metabolites and large-scale biobanks. As exemplars, we cleaned and reformatted more than 594 GWAS traits and pre-calculated the phenotypic correlation matrix for these traits from a large scale “omics” study and UK Biobank (Kettunen *et al*., 2016; Sudlow *et al.,* 2015). In the GitHub repository, we also provide the pre-calculated phenotypic correlation matrix of 221x221 complex human traits in LD Hub. This greatly simplifies the process of multiple testing estimation for these traits.

We now describe some limitations of PhenoSpD, which are general limitations when estimating phenotypic correlation using GWAS summary statistics:

1. One sample setting: the samples of the two GWASs must be from substantially overlapping samples to effectively estimate phenotypic correlation.
2. Genetic or environmental components:
   1. for metaCCA, when genetic components appear to dominate the phenotypic correlation, using beta coefficients to estimate phenotypic correlation will bias the estimation towards the genetic correlation. When the environmental components (“environment” here can be either shared environmental contributions or stochastic phenotypic variation (Davey Smith 2011)) dominate the phenotypic correlation, using beta coefficients to estimate phenotypic correlation will bias estimates towards the null. We consider it can only be applied to the situation in which phenotypic and genetic correlation line up very well, for example metabolites (Würtz P, *et al*., 2014).
   2. For LD score regression, the method is able to capture both genetic correlation (which is represented by the slope of the regression model) and phenotypic correlation (which is represented by the intercept of the regression model). When environmental factors dominate the phenotypic correlation (which means the slope of LD score regression is close to zero), the intercept (which is built up using the error term of the SNP-trait association model) can still reconstruct a substantial component of the phenotypic correlation.
3. Sample size of GWASs: we recommend sample size > 5000 for LD score regression and > 300 for metaCCA
4. Number of SNPs: the number of SNPs included in the model should be more than 200000 to get more accurate correlation estimation.
5. SNP coverage: ideally, SNPs across the whole genome should be included in the model.

**Potential application of PhenoSpD**

The main application of PhenoSpD is to determine the appropriate multiple testing correction for high-dimensional phenotypic data from a single cohort or study (e.g. metabolomics (Shin *et al.,* 2014), epigenetics (Gaunt *et al.,* 2016), transcriptomics (The GTEx Consortium 2013) and proteomics (Suhre *et al.,* 2017) platforms that assay hundreds to thousands of traits). This approach is less stringent than the very conservative Bonferroni correction, which is inappropriate given that many phenotypes are correlated and not actually independent. In an ideal world, if the individual-level data for such studies would be easily and readily available, it would be straightforward to determine the phenotypic correlations by using individual level phenotype data. However, individual-level phenotype data is not as readily available as GWAS summary statistics (which are increasingly openly accessible and downloadable).

Large-scale biobanks, such as UK Biobank (Sudlow *et al.,* 2015), are increasingly measuring a large number of phenotypes in the same sample. It is therefore likely to become more common for large-scale GWAS studies of diverse phenotypes to be published from the same set of participants, in contrast to the current situation of lots of GWAS from different samples with different phenotypic measurements. The proposed method will be particularly applicable for these biobanks. For example, recently automated GWAS of more than 2400 human traits has been performed in UK Biobank, enabling PhenoSpD analysis on a very large number of individuals (data can be downloaded from <http://www.nealelab.is/blog/2017/7/19/rapid-gwas-of-thousands-of-phenotypes-for-337000-samples-in-the-uk-biobank>).

Moreover, PheWAS is becoming a very popular tool and the dimensionality of PheWAS will increase greatly in coming years. We are moving away from single, hypothesis-driven analyses to high dimensional hypothesis-free PheWAS analyses. Tools like PhenoSpD are therefore potentially extremely useful for PheWAS approaches such as MR-PheWAS (Millard *et al.,* 2015) and MR-Base (Hemani *et al.,* 2018). To maximise the value of overlapping samples in published GWAS, we recommended a specific strategy when applying PhenoSpD. The strategy is, correlated traits tend to be measured and studied within the same pool of individuals from a specific consortium. For example, anthropometric traits are mostly meta-analysed by the GIANT consortium (Wood *et al.,* 2014; Locke *et al.,* 2015; Shungin *et al.,* 2015); and most of the glucose and insulin related traits are studied in MAGIC consortium (Scott *et al.,* 2012; Manning *et al.,* 2012; Wheeler *et al.,* 2017). We could estimate the phenotypic correlations inside each consortium. In such way, we will be able to utilise the overlapping samples to reconstruct part of the phenotypic correlation.

In general, with the development of resources like LD Hub and MR-Base and large-scale phenotyping and GWAS in major biobanks (e.g. UK Biobank), the proposed method, PhenoSpD, will become more relevant.

# Availability of Data and Materials

Project name: PhenoSpD

Project home page: <https://github.com/MRCIEU/PhenoSpD>

License: PhenoSpD is licensed under GNU GPL v3.

All data used in this manuscript are publicly available and can be downloaded from the following links.

GWAS results from Shin *et al.,*: <http://mips.helmholtz-muenchen.de/proj/GWAS/gwas/gwas_server/shin_et_al.metal.out.tar.gz>

GWAS results from Kettunen *et al.,*: [http://www.computationalmedicine.fi/data#NMR_GWAS](http://www.computationalmedicine.fi/data#NMR_GWAS)

UK Biobank GWAS results from Neale Lab: <http://www.nealelab.is/blog/2017/7/19/rapid-gwas-of-thousands-of-phenotypes-for-337000-samples-in-the-uk-biobank>.

Operating systems: Linux, OS X, windows

Programming languages: R

Funding

This work was supported by the Medical Research Council (MC_UU_12013/4 and MC_UU_12013/8). This work was in part supported by Cancer Research UK programme grant number C18281/A19169 (the Integrative Cancer Epidemiology Programme). PCH is a Cancer Research UK Population Research Fellow (grant number C52724/A20138). TGR is a UKRI Innovation Research Fellow (MR/S003886/1)

Conflict of Interest: TRG, GDS and GH receive funding from GlaxoSmithKline and Biogen for research unrelated to the work described herein.

References

Bulik-Sullivan, *et al*. (2015a) LD Score Regression Distinguishes Confounding from Polygenicity in Genome-Wide Association Studies. Nat. Genet., 47, 291–295

Bulik-Sullivan. *et al*. (2015b) An atlas of genetic correlations across human diseases and traits. Nat. Genet., 47, 1236–1241.

Cichonska. *et al*. (2016) metaCCA: summary statistics-based multivariate meta-analysis of genome-wide association studies using canonical correlation analysis. Bioinformatics 32 (13): 1981-1989.

Davey Smith G. (2011). Epidemiology, epigenetics and the 'Gloomy Prospect': embracing randomness in population health research and practice. Int J Epidemiol. 2011 Jun;40(3):537-62.

Gaunt TR, *et al*. (2016) Systematic identification of genetic influences on methylation across the human life course. Genome biology 17 (1), 61.

Hemani G, *et al*. (2018) MR-Base: an integrated database and platform for systematic causal inference across the phenome using results from genome-wide association studies. eLife. Accepted.

Kettunen *et al*. (2016) Genome-wide study for circulating metabolites identifies 62 loci and reveals novel systemic effects of LPA. Nat Commun. 7:11122.

Li J, Ji L. (2005) Adjusting multiple testing in multilocus analyses using the eigenvalues of a correlation matrix. Heredity 95:221-227

Locke AE *et al*. (2015) Genetic studies of body mass index yield new insights for obesity biology. Nature 518, 197-206.

Manning AK *et al*. (2012) A genome-wide approach accounting for body mass index identifies genetic variants influencing fasting glycemic traits and insulin resistance. Nat Genet. 13;44(6):659-69.

Millard LA *et al* (2015) MR-PheWAS: hypothesis prioritization among potential causal effects of body mass index on many outcomes, using Mendelian randomization. Sci Rep. 2015 Nov 16;5:16645. doi: 10.1038/srep16645.

Nyholt DR. (2004) A simple correction for multiple testing for SNPs in linkage disequilibrium with each other. Am J Hum Genet 74(4):765-769.

Pasaniuc B, Price AL. (2017) Dissecting the genetics of complex traits using summary association statistics. Nat Rev Genet. 18(2):117-127.

Scott RA *et al*. (2012) Large-scale association analyses identify new loci influencing glycemic traits and provide insight into the underlying biological pathways. Nat Genet. 44(9):991-1005.

Shin SY *et al*. (2014) An atlas of genetic influences on human blood metabolites. Nat Genet. 46(6):543-50.

Shungin D, *et al*. (2015) New genetic loci link adipose and insulin biology to body fat distribution. Nature 518, 187-196.

Sudlow C, *et al*. (2015) UK biobank: an open access resource for identifying the causes of a wide range of complex diseases of middle and old age. PLoS Med. 2015;12(3):e1001779. doi: 10.1371/journal.pmed.1001779.

Suhre K *et al*. (2017) Connecting genetic risk to disease end points through the human blood plasma proteome. Nat Commun. 27;8:14357. doi: 10.1038/ncomms14357.

The GTEx Consortium. (2013) The Genotype-Tissue Expression (GTEx) project. Nature Genetics. 45(6):580-5. doi: 10.1038/ng.2653

Wheeler E *et al*. (2017) Impact of common genetic determinants of Hemoglobin A1c on type 2 diabetes risk and diagnosis in ancestrally diverse populations: A transethnic genome-wide meta-analysis. PLoS Med. 2017 Sep 12;14(9):e1002383.

Wood AR *et al*. (2014). Defining the role of common variation in the genomic and biological architecture of adult human height (2014). Nature Genetics 46, 1173-86.

Würtz P, *et al*. (2014) Metabolic signatures of adiposity in young adults: Mendelian randomization analysis and effects of weight change. PLoS Med. 11(12):e1001765. doi: 10.1371/journal.pmed.1001765. eCollection 2014 Dec.

Zheng J *et al*. (2017) LD Hub: a centralized database and web interface to perform LD score regression that maximizes the potential of summary level GWAS data for SNP heritability and genetic correlation analysis. Bioinformatics. 33 (2): 272-279.
