# Supplementary material for: PhenoSpD: an integrated toolkit for phenotypic correlation estimation and multiple testing correction using GWAS summary statistics
Source: Gigascience. 2018 Aug 24;7(8):giy090. doi: 10.1093/gigascience/giy090 (PMC6109640; doi:10.1093/gigascience/giy090)
Supplement: Response_to_Reviewer_Comments_Original_Submission.pdf [file giy090_response_to_reviewer_comments_original_submission.pdf]

Author's Response To Reviewer Comments

Close

Reply to reviews comments

We created a clean Word file called "Reply-to-reviewers-comments-final.docx" as a supplementary document. To make sure the reviewers can review our reply, we also attached the same reply below.

Reviewer #1: Identifying phenotypic correlations between human traits or diseases is a very important problem. The authors developed PhenoSpD to estimate phenotypic correlation structure by utilizing existing summary statistics based methods (metaCCA or LD score regression) and reduce the multiple testing burden for traits by performing a spectral decomposition (SpD) on the estimated correlation matrix. The authors performed simulation studies to assess the performance of PhenoSpD in diverse simulation scenarios. A comparison between metaCCA and LD score regression was also discussed. Using a real data set, the authors well illustrated the performance and possible applications of PhenSpD. However, I have several concerns that I would like to see discussed.
1. My major concern is that PhenSpD is just using existing methods to infer phenotypic correlation structure (metaCCA and LD score regression) and determine the number of independent tests (SpD). What is the novelty or advantages of this method/software compared to a script executing all three existing tools in the order described in Fig1?
Answer: We appreciated reviewer 1's comments on the novelty of the method. We explained how we use the by-products of the existing methods in the introduction section:
"In this paper, we consider two methods that can be used (but were not designed) to estimate phenotypic correlations using GWAS summary statistics as by-products of the main purposes of those methods. First, MetaCCA (Cichonska et al., 2016) is a multivariate meta-analysis tool that allows multivariate representation of both genotype and phenotype. As a by-product, metaCCA estimates the phenotypic correlation between two traits based on a Pearson correlation between two univariable regression coefficients (betas) across a set of genetic variants. Second, bivariate linkage disequilibrium (LD) score regression (Bulik-Sullivan et al., 2015b) is a state-of-art approach to estimate genetic correlations between a pair of traits. As a consequence, bivariate LD score regression approach allows estimation of phenotypic correlation amongst the overlapping samples of two GWASs. Assume genetic and non-genetic components of two phenotypes are independent, the genetic covariance matrix (build up by the beta coefficients of the genetic association test) will capture the genetic effects, while the error covariance matrix (build up by the error term of the genetic association test) will capture the environmental (non-genetic) effects. Using bi-variate LD score regression model, we will be able to capture both (genetic correlation will be represented by the slope of the regression model and phenotypic correlation will be represented by the intercept of the regression model) (Bulik-Sullivan et al., 2015b)."

We also discussed the novelty and advantages of PhenoSpD in much more details in the discussion section.
"
There are a few key advantages of PhenoSpD. Firstly, our proposed approach utilizes the by-products of two established methods – metaCCA and bivariate LD score regression. We also greatly extended the simulations and real-world application of the by-products of these two methods and made it clear that metaCCA can only be applied to metabolites and

bivariate LD score regression can only be used to estimate phenotypic correlation under certain conditions (Table S1), which added value to the previous findings (Cichonska. et al. 2016; Bulik-Sullivan. et al 2015).

In addition, we provided a simple and user-friendly tool to correct for multiple testing for large scale "omics" data analyses and phenome-wide association studies (PheWAS),. The multiple testing correction will still be stringent (since limited sample overlap between two GWASs will drive phenotypic correlation towards null), but less stringent than Bonferroni correction. This approach is therefore particularly valuable for GWAS of complex human traits such as metabolites and large-scale biobanks. As exemplars, we cleaned and reformatted more than 594 GWAS traits and pre-calculated the phenotypic correlation matrix for these traits from a large scale "omics" study and UK Biobank (Kettunen et al. 2016; Sudlow et al 2015). In the GitHub repository, we also provide the pre-calculated phenotypic correlation matrix of 221x221 complex human traits in LD Hub. This greatly simplified the process of multiple testing estimation for these traits."

2. I am also concerned about the suggested simulation design. While authors simulated diverse variables, they did not simulate important factors in GWASs such as linkage disequilibrium, ethnical diversity/population stratification or number of causal variants. It would be interesting to see the influence of these factors on the accuracy of phenotypic correlation structure estimation.
Answer: Thanks for the suggestion from reviewer 1, we have further estimated the influence of both LD and causal variants to the proposed approach.
We firstly conducted a new set of comparison to estimate the effect of linkage disequilibrium on the accuracy, and we found that SNPs with high LD will reduce the accuracy of the method. We included this simulation in a new group in Table 2 (linkage disequilibrium 1~6).
We also stated this finding in the results section (page 4):
"In addition, we observed that the number of SNPs included in the model will also influence the accuracy of the phenotypic correlation estimation. We also found that if all tested SNPs were from one or few LD blocks (in other words, in high LD with each other), the accuracy of the phenotypic correlation will decrease (Table 2).."

In addition, we appreciate that the review raised the important question of causal variants. We considered different situations and conducted a real case analysis to test the influence of number of causal variants on the estimation of phenotypic correlation. We added the following information in to the methods and results section:
"In addition, we tested whether the number of causal variants (which are tagged by the genetic association signals) may affect the accuracy of the phenotypic correlation using 4 pairs of metabolites from Shin et al. The 4 pairs of metabolites were selected because they have a wide range of observed phenotypic correlation from 0.2 to 0.85. To validate the accuracy, we compared the observed phenotypic correlation with the phenotypic correlation estimated by LD score regression. The key assumption for this validation analysis is polygenicity, which means many genetic variants have small effects on human traits (Figure 3). To consider both number of causal and polygenic variants in this validation, we setup 8 groups of SNPs based on their effects on the traits. The 8 groups were (1) all GWAS SNPs; (2) SNPs with Chi square statistics (square of Z scores) smaller than 40; (3) SNPs with $X2 < 30$; (4) SNPs with $X2 < 20$; (5) SNPs with $X2 < 10$; (6) SNPs with $X2 < 3.84$; (7) SNPs with $X2 < 2.69$ and (8) SNPs with $X2 < 1$. In other words, we reduced the number of casual variants from the model and compared the accuracy of the phenotypic correlation

estimation.”

For population stratification, we further checked the literature and found that the bivariate LD score regression will adjust for population stratification for the estimated correlation. It is less a concern for the proposed approach.

3. It would be helpful for users if the authors can suggest possible downstream analyses using the output (phenotypic correlation matrix and the number of independent traits) of the software.
Answer: We really appreciated that reviewer 1 asked about the possible downstream analyses / applications of the proposed method. In the discussion, we added a new section listed three types of potential downstream analyses using the output of PhenoSpD:
“
The main application of PhenoSpD is to determine the appropriate multiple testing correction for high-dimensional molecular profiling platform on a single cohort (i.e. metabolomics (Shin et al 2014), epigenetics (Gaunt et al 2016), transcriptomics (The GTEx Consortium 2013) and proteomics (Suhre et al 2017) platform that assays hundreds to thousands of entities on sample people). This approach is less stringent than the very conservative Bonferroni correction, which is inappropriate given that multiple analytes are highly correlated and not actually independent. In an ideal world, if the individual-level data for such studies would be easily and readily available the analysis such that it would be straightforward to determine the phenotypic correlations by using individual level phenotype data. However, individual-level phenotype data is not readily available for most such studies whereas GWAS summary statistics from the studies are more and more openly accessible and downloadable.

In addition, more and more large-scale biobanks, such as UK Biobank (Sudlow et al 2015), measured a large number of phenotypes in the same sample. We believe it will be more typical to see large scale GWAS studies of diverse phenotypes from same set of participants in the near future, rather than the current situation of lots of GWAS from different samples with different phenotypic measurements. The proposed method will be particularly applicable for these biobanks. For example, recently automated GWAS of more than 2400 human traits has been performed in UK Biobank release enabling PhenoSpD analysis on a very large number of individuals (data can be downloaded from http://www.nealelab.is/blog/2017/7/19/rapid-gwas-of-thousands-of-phenotypes-for-337000-samples-in-the-uk-biobank).

Moreover, PheWAS is becoming a very popular tool and the dimensionality of PheWAS will increase greatly in coming years. We're moving away from single, hypothesis-driven analyses to high dimensional hypothesis-free PheWAS analyses. Under such trend, we consider PhenoSpD as an extremely useful tool for PheWAS methods such as MR-PheWAS (Millard et al), MR-Base and LD Hub. Till now, the GWAS data from these platforms are from either overlapped or non-overlapped samples. To maximise the value of the overlapped samples within these platforms, we recommended a specific strategy when applying PhenoSpD. The strategy is, correlated traits tends to be measured and studied within the same pool of individuals from a specific consortium. For example, anthropometric traits are mostly meta-analysed by the GIANT consortium (https://portals.broadinstitute.org/collaboration/giant/index.php?title=GIANT_consortium&oldid=251). We could estimate the phenotypic correlations inside each consortium. In such way, we will be able to utilise the overlapped samples to reconstruct part of the phenotypic

correlations in MR-Base and LD Hub.”

4. In page 3 line 40, "Table S2" is the estimated correlation matrix nor 123 metabolites GWAS list used in the study.
Answer: Thanks for the reminder. We have listed the metabolites GWAS list in table Table S3 now.
5. In page 6 line 43, please correct "correlation structure of the correlation structure of ".
Answer: Thanks for the correction, we have changed the section to
“We illustrate the application of PhenoSpD by estimating the phenotypic correlation structure and number of independent tests of 107 metabolites from Kettunen’s study (Kettunen et al, 2016) and 487 UK Biobank traits for the very first time.”
Reviewer #2: Zheng et al. introduce a new R package PhenoSpD that utilizes 3 previously published methods (metaCCA, bivariate LD-score Regression and SNPSpD) in a different context than the 3 were originally developed to address the issue of how to correct for multiple hypothesis testing in the special case of a large number of GWAS performed on the same samples. The main application of PhenoSpD is intended for the study design of running a large molecular profiling platform on a single cohort (i.e. metabolomic/lipidomic/proteomic platform that assays hundreds to thousands of entities on sample people) and trying to determine the appropriate multiple-testing correction instead of the very conservative and strict Bonferroni correction given that multiple analytes are highly correlated and not actually independent. If the individual-level data for such studies were easily and readily available the analysis and determination would be straightforward and has been demonstrated to give robust and insightful results, but individual-level data is not readily available for most such studies whereas summary-statistics from the studies are. Therefore Zheng et al. set-out to provide a tool that can utilize summary-statistics from such a study by leveraging previously worked-out algorithms to enable a robust tool for multiple-testing correction of summary statistics using the phenotypic correlation estimated from either metaCCA or bivariate LD-score Regression and then applying spectral decomposition of that estimated phenotypic correlation matrix to estimate the number of independent tests. The study design that this tool is meant for is limited in scope currently, but with the development of resources like MR-Base and running of large molecular platforms on large biobanks (i.e. UK Biobank) this tool could become more relevant.
Answer: It is our great honor to receive reviewer 2’s comments. Especially their comment that PhenoSpD is valuable for large-scale molecular platforms and large biobanks such as UK biobank. In the discussion section, we added a new section listing the potential applications of PhenoSpD.
“The main application of PhenoSpD is to determine the appropriate multiple testing correction for high-dimensional molecular profiling platform on a single cohort (i.e. metabolomics (Shin et al 2014), epigenetics (Gaunt et al 2016), transcriptomics (The GTEx Consortium 2013) and proteomics (Suhre et al 2017) platform that assays hundreds to thousands of entities on sample people). This approach is less stringent than the very conservative Bonferroni correction, which is inappropriate given that multiple analytes are highly correlated and not actually independent. In an ideal world, if the individual-level data for such studies would be easily and readily available the analysis such that it would be straightforward to determine the phenotypic correlations by using individual level phenotype data. However, individual-level phenotype data is not readily available for most such studies whereas GWAS summary statistics from the studies are more and more openly accessible and downloadable.

In addition, more and more large-scale biobanks, such as UK Biobank (Sudlow et al 2015),

measured a large number of phenotypes in the same sample. We believe it will be more typical to see large scale GWAS studies of diverse phenotypes from same set of participants in the near future, rather than the current situation of lots of GWAS from different samples with different phenotypic measurements. The proposed method will be particularly applicable for these biobanks. For example, recently automated GWAS of more than 2400 human traits has been performed in UK Biobank release enabling PhenoSpD analysis on a very large number of individuals (data can be downloaded from http://www.nealelab.is/blog/2017/7/19/rapid-gwas-of-thousands-of-phenotypes-for-337000-samples-in-the-uk-biobank)."

Q: There are three main limitations in terms of the content of the manuscript, and related to each other, that should be addressed that I'll discuss further below:
(1) a basic discussion of the relationship between genetic correlation and phenotypic correlation, how that was modeled in the simulations and how that impacts the results of the simulations, analysis/results of Shin et al. 2014 data or utilization of metaCCA and bivariate LD-score regression's estimates
(2) there isn't even a cursory overview of the methods that this tool is building upon
(3) there isn't a recognition or discussion (except in passing) that the tool is utilizing consequences/sub-results of metaCCA and bivariate LD-score Regression as opposed to the main purposes of those methods.
Answer: We really appreciate reviewer 2 summing up the key limitations of the manuscript. We have refined the manuscript based on these three points. Details of the refinements are listed below.

Q: Given that this isn't a statistical genetics specific journal there needs to be at least a cursory introduction to the components that make up phenotypic correlation, which roughly speaking for the context of this manuscript and tool are genetic and environmental components. This is extremely important in this context because the tool wants to use genetic component summary statistics to reconstruct total phenotypic correlations, but the summary statistics can't capture the environmental component, and as the environmental component becomes the dominant component the use of genetic component summary statistics will fail to capture the phenotypic correlation. This fact is hinted at on page 2 lines 55-58, "Within each sample, we further assume complex human traits were influenced by both genetic and environmental factors. We simulated the phenotype data of two correlated traits .... varying genetic factors (ranging from 10 to 10,000 SNPs) and 100 environmental factors."
Answer: We agree with reviewer 2's suggestion on introducing the key facts/assumptions to the general audience. We added the following explanation in the introduction section: "Fortunately, we are now in the post genome-wide association study (GWAS) era, in which GWAS summary results are open access and freely available online for a large number of human diseases and traits (Pasaniuc and Price, 2017). It can therefore be valuable to use these genetic association summary statistics to reconstruct total phenotypic correlations across the human phenome. The key facts/assumptions here are: 1) the components that make up phenotypic correlation are the genetic and environmental (non-genetic) components; 2) the genetic association information is able to capture both genetic and non-genetic components of the phenotypic correlation (Bulik-Sullivan et al., 2015b)."

Q: There is no discussion in the text (and not clear to me from the simulation R script provided) of how in the simulation the amount of the phenotypic correlation was assigned to the environmental and genetic components and the effect on the metaCCA results this had.

As a side note, it was only by reading the R script that I knew that the simulations were computed with metaCCA because this is not mentioned in the text although it needs to be.
Answer: We appreciate the reviewer raising this point. We conducted some additional simulations to consider the influence of the amount of the phenotypic correlation that was assigned to the environmental and genetic components:
1. either environmental or genetic components become the dominant components.
2. both genetic and environmental components play a role (10%-90%).
In the simulation section, we added the following paragraph:
“We then assigned the phenotypic correlation to its genetic and environmental components, each of which explained 10% to 90% of the total phenotypic variance. These genetic and environmental components were further assigned to each of the genetic and environmental factors in the model randomly. We also simulated two extreme cases where either the genetic and environmental components dominate the phenotypic correlation.”

We included the result of these additional simulations in Table 1 and Table 2 with row name Genetic_Env_components. We also sum up our findings in the results section:
“In more details, one important question here is how the genetic and environmental factors affect the phenotypic correlation estimation. As show in Table 1, we found that when the environmental components dominate the phenotypic correlation, the metaCCA estimates will bias towards the null. In addition, when genetic component dominates, metaCCA estimates will bias towards the genetic correlation. This fit the assumption that the genetic covariance matrix (build up by the beta coefficients from two GWASs) will only capture the genetic effects. MetaCCA used the beta coefficients to estimate the correlation, which approximately estimate the genetic correlation within the overlapped samples. This is consistent with the simulation results in Table 1.

In contrast to metaCCA, LD score regression estimates both the genetic covariance matrix and the non-genetic covariance matrix (the error variance in the estimates of effects). In other words, given a bi-variate setting (two GWASs), the slope of the LD score regression represents the genetic correlation, while the intercept term of the LD score regression represents the phenotypic correlation. This is consistent with the results in Table 2.”

In addition, metaCCA and LD score regression has been added to the text in the simulation.
“estimated the phenotypic correlation between trait 1 and trait 2 in the overlapped samples using both metaCCA and LD score regression.”

Q: There is also no discussion of whether in the 10 SNP to 10,000 SNP simulations the total genetic component was kept constant or not, and whether all SNPs in the simulation have some effect or just some of the SNPs.
Answer: We appreciated reviewer’s comments on this. In our simulation, we assumed that a large number of SNPs have small effects on the human traits, which is a state-of-the-art assumption within the genetic field.
“The key assumption for this validation analysis is polygenicity, which means many genetic variants have small effects on human traits.”

Q: This gets to the point of not exactly sure what sort of genetic model is being tested in the simulations. I believe all of this is relevant to interpreting figures 3 & 4, and I have a different interpretation than that offered in the manuscript. Figure 3 does show that overall inferring phenotypic correlation from genetic effect summary statistics does very well for most analytes, with slight deviations from the x=y line most likely accounted for by the

environmental component that the GWAS summary statistics can't model. But there are striking deviations from the x=y line especially at the genetically derived phenotypic correlation == 0. The authors state on page 5, lines 10-11 that, "As show in Figure 4, the points with high level of errors (disagreements) are metabolites with limited sample size. The metabolites with limited sample size also have a limited number of sample overlap....". I disagree with the interpretation of Figure 4 in relation to Figure 3, if you look at the spread of the y-axis at the largest sample size (~8K) there are many metabolites with as large errors as samples below 1K and samples of ~3K. This is showing that there are many metabolites in Shin et al. 2014 with a large environmental component that the genetically derived phenotype correlation cannot estimate.
Answer: We agreed with the review's interpretation for the former Figure 3 (now Figure 4). The interpretation fit with our new simulation results well. As an action, we removed the former Figure 4. We also added the following explanation in the results section:
"The exception is that some traits with large observed correlation have estimated correlation towards null. Two possible interpretations of this discrepancy are (1) The phenotypic correlations of some UK Biobank traits were poorly estimated and potentially mis-specified due to limited sample size; (2) due to missingness of the metabolite measurements, the sample overlapped were limited between some UK Biobank traits."

Q: Since the focus of metaCCA and bivariate LD-score Regression papers was not specifically estimating phenotypic correlation, I didn't see any discussion of this in those papers (although might have been in the supplement of metaCCA paper). This also impacts the use of this tool. Therefore, a discussion of the relationship between genotypic and phenotypic correlation is warranted in the paper, a better description of the simulations performed including how much of the phenotypic correlation modeled is partitioned into genetic and environmental components, and finally the impact all this has on the methods used in the tool and how the tool handles that.
Answer: We appreciated the reviewer mentioned this important point. We have added more information in the introduction, simulation and discussion. Please see our answers re this point above.

Q: I'll address limitations (2) and (3) above together here as they are related and are important in regard to estimating phenotypic correlation from genetic effect summary statistics. The first limitation is that both the metaCCA and bivariate LD-score Regression manuscripts are method development papers with many derivations and those derivations are meant to illustrate the main purpose of those methods, which are multi-variate association analysis in the case of metaCCA and genetic correlation estimation in the case of bi-variate LD-score Regression. Therefore, the fact that there isn't a cursory overview of the methods metaCCA and bi-variate LD-score Regression and what components of those methods that PhenoSpD is actually utilizing requires having to read all 3 papers simultaneously to figure out what is actually going on here. The fact that you are actually utilizing a consequence of bivariate LD-score Regression is discussed only in passing on page 6, lines 10-12.
The key point is that you are utilizing the intercept term from Eq. 2 from Bulik-Sullivan et al. 2015b, which will approach zero as sample overlap approaches zero, which does not affect the slope of the line which is the point of bivariate LD-score Regression. The intercept term helps reduce the standard error of the genetic correlation and therefore if known is good to provide a priori to the method.
Answer: This is a really good suggestion. We have rewritten the following paragraph in the introduction section.

“In this paper, we consider two methods that can be used (but were not designed) to estimate phenotypic correlations using GWAS summary statistics as by-products of the main purposes of those methods. First, MetaCCA (Cichonska et al., 2016) is a multivariate meta-analysis tool that allows multivariate representation of both genotype and phenotype. As a by-product, metaCCA estimates the phenotypic correlation between two traits based on a Pearson correlation between two univariable regression coefficients (betas) across a set of genetic variants. Second, bivariate linkage disequilibrium (LD) score regression (Bulik-Sullivan et al., 2015b) is a state-of-art approach to estimate genetic correlations between a pair of traits. As a consequence, bivariate LD score regression approach allows estimation of phenotypic correlation amongst the overlapping samples of two GWASs. Assume genetic and non-genetic components of two phenotypes are independent, the genetic covariance matrix (build up by the beta coefficients of the genetic association test) will capture the genetic effects, while the error covariance matrix (build up by the error term of the genetic association test) will capture the environmental (non-genetic) effects. Using bi-variate LD score regression model, we will be able to capture both (genetic correlation will be represented by the slope of the regression model and phenotypic correlation will be represented by the intercept of the regression model) (Bulik-Sullivan et al., 2015b).”

Q: The authors mention on page 1 line 50 MR-Base and LD-Hub having large samples of harmonized summary statistics as a motivation for PhenoSpD, but because most of the studies in these resources have very little sample overlap the phenotypic correlation estimated from bi-variate LD-score Regression will be ~0 and therefore not useful in PhenoSpD. In fact, a quick and cursory inspection of the phenotypic correlation values provided on the LD-Hub download page (Note: the link provided to the excel file on the GitHub page for PhenoSpD is incorrect and dead) (http://ldsc.broadinstitute.org/lookup/) shows that a majority of phenotypic correlation values are ~0 and therefore of no use in PhenoSpD.
Answer: We agree with the reviewer that the major application of PhenoSpD will be large-scale “omics” studies running on a single cohort or large biobank. Besides this major application, we consider PhenoSpD as a useful tool for MR-Base and LD Hub as well. For example, in a recent manuscript we submitted to BioRxiv (https://www.biorxiv.org/content/biorxiv/early/2017/09/14/189076.1.full.pdf), we applied PhenoSpD to 139 selected diseases and risk factors from MR-Base. We classified these traits into groups (each consortium / study is one group), applied PhenoSpD to each group and sum up the totally number of independent traits. Surprisingly, the total number of tests have been reduced by half (from 139 to 62 tests). In the discussion section, we added one more paragraph to explain this type of application.
“Moreover, PheWAS is becoming a very popular tool and the dimensionality of PheWAS will increase greatly in coming years. We're moving away from single, hypothesis-driven analyses to high dimensional hypothesis-free PheWAS analyses. Under such trend, we consider PhenoSpD as an extremely useful tool for PheWAS methods such as MR-PheWAS (Millard et al), MR-Base and LD Hub. Till now, the GWAS data from these platforms are from either overlapped or non-overlapped samples. To maximise the value of the overlapped samples within these platforms, we recommended a specific strategy when applying PhenoSpD. The strategy is, correlated traits tends to be measured and studied within the same pool of individuals from a specific consortium. For example, anthropometric traits are mostly meta-analysed by the GIANT consortium (https://portals.broadinstitute.org/collaboration/giant/index.php?title=GIANT_consortium&oldid=251). We could estimate the phenotypic correlations inside each consortium. In such way, we will be able to utilise the overlapped samples to reconstruct part of the phenotypic

correlations in MR-Base and LD Hub."

Q: To the authors credit they do briefly discuss the limitation of using bivariate LD-score regression, but I believe that a more thorough comparison and recommendation between metaCCA and bivariate LD-score regression is warranted. The same theme is repeated for metaCCA, metaCCA estimates a phenotypic correlation matrix from the genetic effects in order to allow it to perform association testing via Canonical Correlation Analysis on the full covariance matrix after applying a shrinkage algorithm to ensure that is positive semidefinite (PSD). I'm assuming based on the simulation R script and because of the spectral decomposition step that PhenoSpD is using the raw, estimated phenotypic correlation matrix from metaCCA but the authors should state this just for completeness.
Answer: Thanks for reviewer's comments on this. To make the statement complete, we have added the following sentence to the method section.
"The observed phenotypic correlations and correlations estimated by LD score regression were used as input for the SpD approach."

Q: In the metaCCA paper in the method assessment section it states that they only used Chr1 SNPs for calculation of the phenotypic correlation matrix, is the same done in PhenoSpD or does it use the full genome-wide summary statistics?
Answer: Thank you for the reminder from reviewer 2. We included one more column in Table 2 to state clear that we used the SNPs from the whole genome. We also added the following suggestion in the results section.
"In addition, we observed that the number of SNPs included in the model will also influence the accuracy of the phenotypic correlation estimation. We also found that if all tested SNPs were from one or few LD blocks (in other words, in high LD with each other), the accuracy of the phenotypic correlation will decrease (Table 2). Based on these two observations, we recommend including SNPs as wide genomic regions as possible to maximize the accuracy of the estimation."

Q: All of this is to say that in order for this manuscript to fully describe its tool it needs to discuss briefly the methods that it is utilizing, why those methods were originally developed and the assumptions and caveats those methods have in relation to how PhenoSpD is utilizing them. This links to the discussion above between the relationship between phenotypic correlation and genotypic correlation and impact on PhenoSpD's results.
Answer: Thank you for the suggestion from reviewer 2. We included additional discussion of the proposed method, how we utilized the existing methods in this paper and a summary of the simulation findings.
"There are a few key advantages of PhenoSpD. Firstly, our proposed approach utilizes the by-products of two established methods – metaCCA and bivariate LD score regression. We also greatly extended the simulations and real-world application of the by-products of these two methods and made it clear that metaCCA can only be applied to metabolites and bivariate LD score regression can only be used to estimate phenotypic correlation under certain conditions (Table S1), which added value to the previous findings (Cichonska. et al. 2016; Bulik-Sullivan. et al 2015).

In addition, we provided a simple and user-friendly tool to correct for multiple testing for large scale "omics" data analyses and phenome-wide association studies (PheWAS),. The multiple testing correction will still be stringent (since limited sample overlap between two GWASs will drive phenotypic correlation towards null), but less stringent than Bonferroni correction. This approach is therefore particularly valuable for GWAS of complex human

traits such as metabolites and large-scale biobanks. As exemplars, we cleaned and reformatted more than 594 GWAS traits and pre-calculated the phenotypic correlation matrix for these traits from a large scale “omics” study and UK Biobank (Kettunen et al. 2016; Sudlow et al 2015). In the GitHub repository, we also provide the pre-calculated phenotypic correlation matrix of 221x221 complex human traits in LD Hub. This greatly simplified the process of multiple testing estimation for these traits.

We now describe some limitations of PhenoSpD, which are general limitations when estimating phenotypic correlation using GWAS summary statistics:
1. One sample setting: the samples of the two GWASs are from highly overlapped samples.
2. Genetic or environmental components:
a. for metaCCA, when genetic components appear to dominate the phenotypic correlation, using beta coefficients to estimate phenotypic correlation will bias the estimation towards the genetic correlation. When the environmental components (“environment” here can be both shared environmental contributions and stochastic phenotypic variation (Davey Smith 2011)) dominate the phenotypic correlation, using beta coefficients to estimate phenotypic correlation will bias estimates towards the null. We consider it can only be applied to the situation in which phenotypic and genetic correlation line up very well, for example metabolites (Würtz P, et al. 2014).
b. For LD score regression, the method is able to capture both genetic correlation (which is represented by the slope of the regression model) and phenotypic correlation (which is represented by the intercept of the regression model). When environmental factors dominate the phenotypic correlation (which means the slope of LD score regression is close to zero), the intercept (which is built up using the error term of the SNP-trait association model) can still reconstruct a substantial component of the phenotypic correlation.
3. Sample size of GWASs: we recommend sample size > 5000 for LD score regression and > 300 for metaCCA
4. Number of SNPs: the number of SNPs included in the model should be more than 200000 to get more accurate correlation estimation.
5. SNP coverage: ideally, SNPs across the whole genome should be included in the model.”

Q: The real point of all this comes down to the final sentence of the manuscript on Page 6, lines 54-55 "For limitation, PhenoSpD can only be used for human traits from the same sample, which is a general limitation of estimating phenotypic correlation using GWAS summary statistics." Therefore, PhenoSpD will not be a tool that will make great use of the summary statistics in MR-Base and LD-Hub because of the limited sample overlap from the studies in those resources. PhenoSpD provides a useful tool in the case of large metabolomic/lipidomic/proteomic platforms run on a single cohort, and useful for those groups who only have access to the summary statistics as opposed to the groups originally generating and analyzing the data.
Answer: We really appreciate reviewer 2 recapping the limitation of PhenoSpD here. Please see the answer we have given above re this point.

Q: Quick Notes on GitHub Repository:
- As mentioned above the link to the LD Hub excel spreadsheet is incorrect and dead
- Would be good to provide an example of the output as well as the input files
Answer: Thank you for checking the GitHub repository. We have corrected the LD Hub link and provided examples for the input and output files. We added the example input files to the repository. The GWAS input example is called “PhenoSpD_input_example.txt” and the

phenotypic correlation input example is called
“LD-Hub_phenotypic_correlation_221x221.txt”.

Close
