# Supplementary material for: PhenoSpD: an integrated toolkit for phenotypic correlation estimation and multiple testing correction using GWAS summary statistics
Source: Gigascience. 2018 Aug 24;7(8):giy090. doi: 10.1093/gigascience/giy090 (PMC6109640; doi:10.1093/gigascience/giy090)
Supplement: Reviewer_1_Report_(Original_Submission) -- Donghyung Lee — 8/29/2017 Reviewed [file giy090_reviewer_1_report_(original_submission).pdf]

Reviewer Report

Title: **PhenoSpD: an integrated toolkit for phenotypic correlation estimation and multiple testing correction using GWAS summary statistics**

Version: **Original Submission** **Date:** 8/29/2017

Reviewer name: **DONGHYUNG LEE**

Reviewer Comments to Author:

Identifying phenotypic correlations between human traits or diseases is a very important problem. The authors developed PhenoSpD to estimate phenotypic correlation structure by utilizing existing summary statistics based methods (metaCCA or LD score regression) and reduce the multiple testing burden for traits by performing a spectral decomposition (SpD) on the estimated correlation matrix. The authors performed simulation studies to assess the performance of PhenoSpD in diverse simulation scenarios. A comparison between metaCCA and LD score regression was also discussed. Using a real data set, the authors well illustrated the performance and possible applications of PhenSpD. However, I have several concerns that I would like to see discussed. 1. My major concern is that PhenSpD is just using existing methods to infer phenotypic correlation structure (metaCCA and LD score regression) and determine the number of independent tests (SpD). What is the novelty or advantages of this method/software compared to a script executing all three existing tools in the order described in Fig1?2. I am also concerned about the suggested simulation design. While authors simulated diverse variables, they did not simulate important factors in GWASs such as linkage disequilibrium, ethnical diversity/population stratification or number of causal variants. It would be interesting to see the influence of these factors on the accuracy of phenotypic correlation structure estimation.3. It would be helpful for users if the authors can suggest possible downstream analyses using the output (phenotypic correlation matrix and the number of independent traits) of the software. 4. In page 3 line 40, "Table S2" is the estimated correlation matrix nor 123 metabolites GWAS list used in the study.5. In page 6 line 43, please correct "correlation structure of the correlation structure of ".

Level of Interest

Please indicate how interesting you found the manuscript: An article of limited interest

Quality of Written English

Please indicate the quality of language in the manuscript: Acceptable

Declaration of Competing Interests

Please complete a declaration of competing interests, considering the following questions:

- Have you in the past five years received reimbursements, fees, funding, or salary from an organisation that may in any way gain or lose financially from the publication of this manuscript, either now or in the future?
- Do you hold any stocks or shares in an organisation that may in any way gain or lose financially from the publication of this manuscript, either now or in the future?

- Do you hold or are you currently applying for any patents relating to the content of the manuscript?
- Have you received reimbursements, fees, funding, or salary from an organization that holds or has applied for patents relating to the content of the manuscript?
- Do you have any other financial competing interests?
- Do you have any non-financial competing interests in relation to this paper?

If you can answer no to all of the above, write 'I declare that I have no competing interests' below. If your reply is yes to any, please give details below.

I declare that I have no competing interests.

I agree to the open peer review policy of the journal. I understand that my name will be included on my report to the authors and, if the manuscript is accepted for publication, my named report including any attachments I upload will be posted on the website along with the authors' responses. I agree for my report to be made available under an Open Access Creative Commons CC-BY license (http://creativecommons.org/licenses/by/4.0/). I understand that any comments which I do not wish to be included in my named report can be included as confidential comments to the editors, which will not be published.

I agree to the open peer review policy of the journal

To further support our reviewers, we have joined with Publons, where you can gain additional credit to further highlight your hard work (see: https://publons.com/journal/530/gigascience). On publication of this paper, your review will be automatically added to Publons, you can then choose whether or not to claim your Publons credit. I understand this statement. Yes
