# Supplementary material for: PhenoSpD: an integrated toolkit for phenotypic correlation estimation and multiple testing correction using GWAS summary statistics
Source: Gigascience. 2018 Aug 24;7(8):giy090. doi: 10.1093/gigascience/giy090 (PMC6109640; doi:10.1093/gigascience/giy090)
Supplement: Reviewer_2_Report_(Original_Submission) -- Aaron Day-Williams — 9/8/2017 Reviewed [file giy090_reviewer_2_report_(original_submission).pdf]

Reviewer Report

Title: **PhenoSpD: an integrated toolkit for phenotypic correlation estimation and multiple testing correction using GWAS summary statistics**

Version: **Original Submission** **Date:** 9/8/2017

Reviewer name: **Aaron Day-Williams**

Reviewer Comments to Author:

Zheng et al. introduce a new R package PhenoSpD that utilizes 3 previously published methods (metaCCA, bivariate LD-score Regression and SNPSpD) in a different context than the 3 were originally developed to address the issue of how to correct for multiple hypothesis testing in the special case of a large number of GWAS performed on the same samples. The main application of PhenoSpD is intended for the study design of running a large molecular profiling platform on a single cohort (i.e. metabolomic/lipidomic/proteomic platform that assays hundreds to thousands of entities on sample people) and trying to determine the appropriate multiple-testing correction instead of the very conservative and strict Bonferroni correction given that multiple analytes are highly correlated and not actually independent. If the individual-level data for such studies were easily and readily available the analysis and determination would be straightforward and has been demonstrated to give robust and insightful results, but individual-level data is not readily available for most such studies whereas summary-statistics from the studies are. Therefore Zheng et al. set-out to provide a tool that can utilize summary-statistics from such a study by leveraging previously worked-out algorithms to enable a robust tool for multiple-testing correction of summary statistics using the phenotypic correlation estimated from either metaCCA or bivariate LD-score Regression and then applying spectral decomposition of that estimated phenotypic correlation matrix to estimate the number of independent tests. The study design that this tool is meant for is limited in scope currently, but with the development of resources like MR-Base and running of large molecular platforms on large biobanks (i.e. UK Biobank) this tool could become more relevant.There are three main limitations in terms of the content of the manuscript, and related to each other, that should be addressed that I'll discuss further below: (1) a basic discussion of the relationship between genetic correlation and phenotypic correlation, how that was modeled in the simulations and how that impacts the results of the simulations, analysis/results of Shin et al. 2014 data or utilization of metaCCA and bivariate LD-score regression's estimates (2) there isn't even a cursory overview of the methods that this tool is building upon(3) there isn't a recognition or discussion (except in passing) that the tool is utilizing consequences/sub-results of metaCCA and bivariate LD-score Regression as opposed to the main purposes of those methods.Given that this isn't a statistical genetics specific journal there needs to be at least a cursory introduction to the components that make up phenotypic correlation, which roughly speaking for the context of this manuscript and tool are genetic and environmental components. This is extremely important in this context because the tool wants to use genetic component summary statistics to reconstruct total phenotypic correlations but the summary statistics can't capture the environmental component, and as the environmental component becomes the dominant component the use of genetic component summary statistics will fail to capture the phenotypic correlation. This fact is hinted at on page 2 lines 55-58, "Within each sample, we further assume complex human traits were influenced by both genetic and environmental factors. We simulated the phenotype data of two correlated traits .... varying genetic factors (ranging from 10 to 10,000 SNPs) and 100 environmental factors." There is no discussion in the text (and not clear to me from the simulation R script provided) of how in the simulation the amount of the phenotypic correlation was assigned to the environmental and genetic components and the effect on the metaCCA results this had. As a side note, it was only by reading the R script that I knew that the simulations were computed with metaCCA because this is not mentioned in the text although it needs to be. There is also no discussion of whether in the 10 SNP to 10,000 SNP simulations the total genetic component was kept constant or not, and whether all SNPs in the simulation have some effect or just some of the SNPs. This gets to the point of not exactly sure what sort of genetic model is being tested in the simulations. I believe all of this is relevant to interpreting figures 3 & 4, and I

have a different interpretation than that offered in the manuscript. Figure 3 does show that overall inferring phenotypic correlation from genetic effect summary statistics does very well for most analytes, with slight deviations from the x=y line most likely accounted for by the environmental component that the GWAS summary statistics can't model. But there are striking deviations from the x=y line especially at the genetically derived phenotypic correlation == 0. The authors state on page 5, lines 10-11 that, "As show in Figure 4, the points with high level of errors (disagreements) are metabolites with limited sample size. The metabolites with limited sample size also have a limited number of sample overlap....". I disagree with the interpretation of Figure 4 in relation to Figure 3, if you look at the spread of the y-axis at the largest sample size (~8K) there are many metabolites with as large errors as samples below 1K and samples of ~3K. This is showing that there are many metabolites in Shin et al. 2014 with a large environmental component that the genetically derived phenotype correlation cannot estimate. Since the focus of metaCCA and bivariate LD-score Regression papers was not specifically estimating phenotypic correlation, I didn't see any discussion of this in those papers (although might have been in the supplement of metaCCA paper). This also impacts the use of this tool. Therefore a discussion of the relationship between genotypic and phenotypic correlation is warranted in the paper, a better description of the simulations performed including how much of the phenotypic correlation modeled is partitioned into genetic and environmental components, and finally the impact all this has on the methods used in the tool and how the tool handles that. A grammar not, page 5 line 10 should read "As shown in Figure 4".I'll address limitations (2) and (3) above together here as they are related, and are important in regards to estimating phenotypic correlation from genetic effect summary statistics. The first limitation is that both the metaCCA and bivariate LD-score Regression manuscripts are method development papers with many derivations and those derivations are meant to illustrate the main purpose of those methods, which are multi-variate association analysis in the case of metaCCA and genetic correlation estimation in the case of bi-variate LD-score Regression. Therefore the fact that there isn't a cursory overview of the methods metaCCA and bi-variate LD-score Regression and what components of those methods that PhenoSpD is actually utilizing requires having to read all 3 papers simultaneously to figure out what is actually going on here. The fact that you are actually utilizing a consequence of bivariate LD-score Regression is discussed only in passing on page 6, lines 10-12. The key point is that you are utilizing the intercept term from Eq. 2 from Bulik-Sullivan et al. 2015b, which will approach zero as sample overlap approaches zero, which does not affect the slope of the line which is the point of bivariate LD-score Regression. The intercept term helps reduce the standard error of the genetic correlation and therefore if known is good to provide a priori to the method. The authors mention on page 1 line 50 MR-Base and LD-Hub having large samples of harmonized summary statistics as a motivation for PhenoSpD, but because most of the studies in these resources have very little sample overlap the phenotypic correlation estimated from bi-variate LD-score Regression will be ~0 and therefore not useful in PhenoSpD. In fact, a quick and cursory inspection of the phenotypic correlation values provided on the LD-Hub download page (Note: the link provided to the excel file on the GitHub page for PhenoSpD is incorrect and dead)(http://ldsc.broadinstitute.org/lookup/) shows that a majority of phenotypic correlation values are ~0 and therefore of no use in PhenoSpD. To the authors credit they do briefly discuss the limitation of using bivariate LD-score Regression but I believe that a more thorough comparison and recommendation between metaCCA and bivariate LD-score regression is warranted. The same theme is repeated for metaCCA, metaCCA estimates a phenotypic correlation matrix from the genetic effects in order to allow it to perform association testing via Canonical Correlation Analysis on the full covariance matrix after applying a shrinkage algorithm to ensure that is is postive semidefinite (PSD). I'm assuming based on the simulation R script and because of the spectral decomposition step that PhenoSpD is using the raw, estimated phenotypic correlation matrix from metaCCA but the authors should state this just for completeness. In the metaCCA paper in the method assessment section it states that they only used Chr1 SNPs for calculation of the phenotypic correlation matrix, is the same done in PhenoSpD or does it use the full genome-wide summary statistics? All of this is to say that in order for this manuscript to fully describe its tool it needs to discuss briefly the methods that it is utilizing, why those methods were originally developed and the assumptions and caveats those methods have in relation to how PhenoSpD is utilizing them. This links to the discussion above between the relationship between phenotypic correlation and genotypic correlation and impact on PhenoSpD's results.The real point of all this comes down to the final sentence of the manuscript on Page 6, lines 54-55 "For limitation, PhenoSpD can only be used for human traits from the same sample, which is a general limitation of estimating phenotypic correlation using GWAS summary statistics." Therefore,

PhenoSpD will not be a tool that will make great use of the summary statistics in MR-Base and LD-Hub because of the limited sample overlap from the studies in those resources. PhenoSpD provides a useful tool in the case of large metabolomic/lipidomic/proteomic platforms run on a single cohort, and useful for those groups who only have access to the summary statistics as opposed to the groups originally generating and analyzing the data.Quick Notes on GitHub Repository:- As mentioned above the link to the LD Hub excel spreadsheet is incorrect and dead- Would be good to provide an example of the output as well as the input files

Level of Interest

Please indicate how interesting you found the manuscript: An article whose findings are important to those with closely related research interests

Quality of Written English

Please indicate the quality of language in the manuscript: Acceptable

Declaration of Competing Interests

Please complete a declaration of competing interests, considering the following questions:

- Have you in the past five years received reimbursements, fees, funding, or salary from an organisation that may in any way gain or lose financially from the publication of this manuscript, either now or in the future?
- Do you hold any stocks or shares in an organisation that may in any way gain or lose financially from the publication of this manuscript, either now or in the future?
- Do you hold or are you currently applying for any patents relating to the content of the manuscript?
- Have you received reimbursements, fees, funding, or salary from an organization that holds or has applied for patents relating to the content of the manuscript?
- Do you have any other financial competing interests?
- Do you have any non-financial competing interests in relation to this paper?

If you can answer no to all of the above, write 'I declare that I have no competing interests' below. If your reply is yes to any, please give details below.

I declare that I have no competing interests

I agree to the open peer review policy of the journal. I understand that my name will be included on my report to the authors and, if the manuscript is accepted for publication, my named report including any attachments I upload will be posted on the website along with the authors' responses. I agree for my report to be made available under an Open Access Creative Commons CC-BY license (http://creativecommons.org/licenses/by/4.0/). I understand that any comments which I do not wish to

be included in my named report can be included as confidential comments to the editors, which will not be published.

I agree to the open peer review policy of the journal

To further support our reviewers, we have joined with Publons, where you can gain additional credit to further highlight your hard work (see: https://publons.com/journal/530/gigascience). On publication of this paper, your review will be automatically added to Publons, you can then choose whether or not to claim your Publons credit. I understand this statement. Yes
